# Multi-omics identifies oxidative stress, prothrombotic pathways, and lactoperoxidase variants as key factors in COVID-19 severity

**DOI:** 10.1016/j.ebiom.2025.106111

**Published:** 2026-01-06

**Authors:** Claudio Cappadona, Valeria Rimoldi, Francesca Tettamanzi, Giulia Cardamone, Alberto Mantovani, Giulia Soldà, Elvezia Maria Paraboschi, Rosanna Asselta

**Affiliations:** a Department of Biomedical Sciences, Humanitas University, Via Rita Levi Montalcini 4, 20072, Pieve Emanuele, Milan, Italy; b IRCCS Humanitas Research Hospital, Via Manzoni 56, 20089, Rozzano, Milan, Italy; c William Harvey Research Institute, Queen Mary University, London, EC1M 6BQ, UK

**Keywords:** COVID-19, Multi-omics, Oxidative stress, Lactoperoxidase, Thrombosis, Immune response

## Abstract

**Background:**

Severe acute respiratory syndrome coronavirus 2 (SARS-CoV-2) infected over 26 million individuals in Italy, resulting in ∼200,000 COVID-19-related deaths. Unravelling host genetic factors underlying disease severity is key to understanding progression mechanisms.

**Methods:**

We applied multi-omics approaches to investigate genetic susceptibility to COVID-19 severity in the Italian population. We combined an exome-wide case–control study of rare germline variants (215 severe/critically ill patients vs 1755 controls) with transcriptomic (differential gene expression and alternative splicing) analyses of 59 hospitalised patients to identify signatures associated with severe respiratory outcomes (ICU admission).

**Findings:**

Rare variant analysis revealed significant associations with genes implicated in oxidative stress and mitochondrial dysfunction, including *MTERF1* (FDR = 7.69 × 10^−5^), *TDP1* (FDR = 3.23 × 10^−7^), and *LPO* (FDR = 1.58 × 10^−2^). Pathway analyses confirmed enrichment in “reactive oxygen species”, “oxidative phosphorylation”, and “inflammatory response” pathways. Transcriptomics showed a proinflammatory profile in hospitalised patients (N = 24) and a prothrombotic signature in ICU-admitted individuals (N = 35), reflecting disease progression. Genomic and transcriptomic data integration highlighted *LPO*, encoding the antimicrobial enzyme lactoperoxidase, as the only gene both significantly enriched for damaging variants and upregulated in ICU-admitted cases (log_2_FC = 0.57, FDR = 0.028). Notably, we confirmed the genetic association with severity in independent cohorts (1873 cases vs 508,532 controls; meta-analysis p = 0.0050, OR = 3.44, 95% CI = 1.71–6.89). We propose that *LPO* haploinsufficiency may impair host capacity to neutralise ROS, contributing to COVID-19 progression.

**Interpretation:**

In conclusion, our multi-omics analysis implicates oxidative stress and mitochondrial dysfunction as central to COVID-19 severity, identifying *LPO* as a candidate susceptibility gene.

**Funding:**

Banca Intesa San Paolo, EU Next-Generation EU-MUR-PNRR (INF-ACT, PE00000007), Dolce & Gabbana.

**Research in context**

**Evidence before this study**

Several genome-wide association studies (GWAS) and meta-analyses on common genetic variants indicated that host genetics significantly contribute to susceptibility and severity of COVID-19. However, a comprehensive understanding of the factors that modulate the acute response in severely ill COVID-19 patients, particularly in relation to rare genetic variants and gene expression profiles, remained incomplete. Previous studies already associated certain genes involved in type I and III interferon-mediated immunity with severe COVID-19 outcomes. Additionally, the pathological progression of the disease has been clinically described in three sequential phases (pulmonary, proinflammatory, and prothrombotic) characterised by a hyper-exaggerated host response. This evidence lacked a deep, multi-omics integration to identify specific biological pathways and genes that could predispose individuals to critical illness.

**Added value of this study**

This study adds significant value by performing a comprehensive multi-omics analysis, integrating rare genetic variants (detected by whole-exome sequencing) with transcriptomic profiles (both differential gene expression and alternative splicing patterns, detected by RNA-sequencing) from a cohort of Italian patients with COVID-19. This integrated approach allowed for a deeper and not-conventional exploration of the molecular mechanisms underlying disease severity. Our study identified that rare variants in genes related to oxidative stress, specifically in the reactive oxygen species metabolic pathway, are strongly associated with a more severe COVID-19 phenotype. Furthermore, it revealed that critically severe patients exhibit a distinct prothrombotic transcriptomic profile, characterised by the upregulation of genes related to haemostasis and platelet activation. Most importantly, by integrating both genetic and transcriptomic data, the study identified lactoperoxidase (*LPO*) as a key gene, enriched with rare variants in patients and significantly upregulated in critically ill individuals. This finding was independently validated in a large external cohort (RGC browser), confirming the role of *LPO* beyond the initial cohort. Lastly, the study provides insights into the role of alternative splicing of transcription factors and their impact on gene expression, which was not thoroughly explored in previous research.

**Implications of all the available evidence**

The findings of this study, combined with existing evidence, have important implications for both clinical practice and future research. The strong association of rare variants in oxidative stress-related pathways with COVID-19 severity suggests that impaired host responses to oxidative stress represent a key driver of disease progression. This is highly relevant to human health as it points to potential biomarkers for identifying individuals at a higher risk of developing a severe form of the disease. The discovery of *LPO* as a central player, with both genetic and transcriptomic evidence supporting its role, provides a clear target for further investigation. The impairment of the LPO system, potentially due to rare inactivating variants, could explain the progression to multi-organ failure. Future research should focus on investigating the functional impact of these rare variants on LPO activity and exploring whether therapeutic strategies targeting oxidative stress and the LPO system could mitigate disease severity. Last, the discovery that alternative splicing of transcription factors contributes to the dysregulated transcriptome of critically ill patients provides an additional layer for interpreting the complex disease mechanisms, suggesting that post-transcriptional regulation plays a crucial role in COVID-19 pathophysiology.

## Introduction

Severe acute respiratory syndrome coronavirus 2 (SARS-CoV-2)^1^ is the virus responsible for the development of a respiratory illness, first identified during late 2019 in China^2^ and called coronavirus disease 2019 (COVID-19).^3^ Since then, the virus has rapidly spread to the rest of the worldwide population, primarily through respiratory droplets when an infected person coughs, sneezes, or talks. On March 11, 2020, the World Health Organization (WHO) declared the pandemic.^4^ During the same period, Italy faced a peak in confirmed COVID-19 cases and on March 16, 2020, the country showed the highest estimated case fatality rate in the world, becoming one of the epicentres of the pandemic.^5^

COVID-19 clinical presentation can be extremely heterogeneous, ranging from the absence of symptoms to mild manifestations (including fever, cough, fatigue, and breathing difficulty), up to severe and life-threatening conditions (such as pneumonia with hypoxaemia, acute respiratory distress syndrome, sepsis, and multi-organ failure) observed in 10–20% of symptomatic cases.^6^ The pathological progression of COVID-19 can be classified by three sequential and partially overlapping stages, characterised both by the viral activity and the exacerbated host response: pulmonary, proinflammatory, and prothrombotic.^7^^,^^8^ During the pulmonary phase, SARS-CoV-2 infects the host cells by binding the ACE2 receptor, impairing its role in regulating the renin-angiotensin system (RAS), and starting an acute respiratory distress syndrome (ARDS). In the proinflammatory phase, the overproduction of proinflammatory cytokines unleashes a cytokine storm and overall systemic inflammation, leading to an acute lung injury (ALI). Finally, during the prothrombotic phase, increased platelet aggregation due to neutrophil extracellular traps (NETs) overproduction promotes thrombosis, coagulopathy, and multi-organ failure (MOF).^7^^,^^8^

COVID-19 heterogeneous symptoms can be explained by both intrinsic characteristics of the virus and genetic factors of the infected individual: major risk factors have been identified in the patient age >65 years, male sex, and the presence of comorbidities such as hypertension, diabetes, chronic pulmonary disease, immunodeficiency, cancer, cardiovascular issues, and obesity.^9^^,^^10^ Additionally, environmental and social factors, as well as the genetics of the infected individual likely contribute to the risk of developing severe COVID-19. Several genome-wide association studies (GWAS) and meta-analyses focusing on common variants have shown that host genetic predisposition can significantly contribute to COVID-19 severity and susceptibility.^11^, ^12^, ^13^, ^14^, ^15^, ^16^, ^17^, ^18^ However, a thorough understanding of the factors modulating the acute response in patients with severe COVID-19 is still lacking.

Here, we provide further insights on this specific aspect of the disease by performing a comprehensive multi-omics analysis, encompassing the study of rare genetic variants and gene expression profiling (including alternative splicing, AS, and transcription factor activity).

## Methods

### Study cohorts

The case cohort was composed of 215 (151 males and 64 females; all Italians of Caucasian ancestry) unvaccinated patients hospitalised (in general wards or in intensive care units, ICUs) at the IRCCS Humanitas Research Hospital (ICH) during 2020. COVID-19 severity was defined by hospitalisation and respiratory failure (requiring supplemental oxygen therapy, non-invasive ventilation, invasive mechanical ventilation, or ExtraCorporeal Membrane Oxygenation, ECMO), together with a positive SARS-CoV-2 viral RNA PCR test from nasopharyngeal swabs. For transcriptomic analyses, the RNA of a subset of 59 patients was analysed: 24 only needed supplemental oxygen support or non-invasive ventilation (hereafter referred to as “Hospitalised”), while 35 required invasive mechanical ventilation (intubation with or without ECMO, hereafter referred to as “ICU-admitted”).

The control cohort for the genetic study was composed of 1755 unvaccinated individuals (1552 males and 203 females; all Italians of Caucasian ancestry) from the Atherosclerosis, Thrombosis, and Vascular Biology Italian Study Group, recruited between 1998 and 2001 from 94 Italian hospitals.^19^ Their COVID-19 status is unknown, and thus they represent the Italian general population.

In all cohorts, gender was self-reported and biological sex was verified by genetic data.

### Ethics

The study was approved by the ICH ethics committee (cases, reference number 316/20) and the Ethics Committee of Ospedale Niguarda, Ca’ Granda, on 03/09/1998, under protocol number 4272/98 (control cohort). For the control cohort, subsequent amendments to the protocol have been reviewed and approved by the Comitato Etico Area Vasta Emilia Nord. The study was conducted according to the Declaration of Helsinki. Participants provided written informed consent. For severely ill patients, the ethics committee granted an exemption, allowing the use of fully anonymised surplus material from diagnostic venipuncture.

### Whole-exome sequencing (WES)

For the case cohort, DNA was extracted from whole blood using the Promega Maxwell RSC Blood DNA Kit (Promega, Madison, Wisconsin, USA) on a benchtop automated extractor (Maxwell RSC Instrument, RRID:SCR_025867). Library preparation was performed using the SureSelect Human All Exon V7 kit (Agilent Technologies, Santa Clara, California, USA) according to the manufacturers’ instructions. Whole-exome paired-end 150-nucleotide sequencing was performed on the Illumina NovaSeq 6000 Sequencing System (RRID:SCR_016387; Illumina, San Diego, California, USA).

For the control cohort, genomic DNA was isolated from whole blood using the standard salting-out extraction method. Library construction and in-solution hybrid selection were performed as described previously.^20^^,^^21^ WES was performed on the Genome AnalyzerII, using the v3 or v4 Sequencing-by-Synthesis kit (Illumina) to produce paired-end 76-nucleotide reads.

Sequencing reads from both cohorts were mapped to the reference human genome (GRCh38/hg38) using the Burrows-Wheeler Aligner^22^ (BWA, RRID:SCR_010910, v0.7.15). Duplicated reads, either due to PCR or optical duplication, were removed using Picard (RRID:SCR_006525) MarkDuplicates (v2.18). All exons and their flanking 50 base pairs of intronic sequence were considered for this analysis. Before calling germline variants, the quality score of bases in known sites of variation was recalibrated with Genome Analysis Toolkit^23^ (GATK, RRID:SCR_001876) ApplyBQSR (v4.0.10) using dbSNP^24^ as reference (v138). Cram^25^ files were then exported using SAMtools (v1.11, RRID:SCR_005227) and for each sample, the recalibrated BAM mapping file was used to perform local de-novo assembly of haplotypes using GATK HaplotypeCaller and identify germline variants. Information for each site was stored in intermediate GVCF files, which were used to perform a joint genotyping on all samples from both cohorts using GATK GenomicsDBImport and GenotypeGVCFs. Site-level information without genotyping data were extracted from the VCF file using GATK MakeSitesOnlyVcf and employed with GATK VariantRecalibrator to train a Gaussian mixture model to obtain recalibrated per-variant probabilities, using as “true polymorphic sites” those included in HapMap,^26^ Illumina Omni 2.5 M SNP, 1000 genomes^27^ and dbSNP.

Multiallelic sites were demultiplexed into biallelic sites using BCFtools v1.13 (RRID:SCR_005227). At the sample level, genotypes with Genotype Quality (GQ) > 20 and supported by at least 10 reads (depth of coverage; DP) were kept, while at the site level, only sites with mean DP ≥ 20 and with missing data not greater than 10% were retained. VCFtools v0.1.16 (RRID:SCR_001235) was used to produce per-site and per-individual coverage metrics.

Variants were annotated offline using the Ensembl Variant Effect Predictor^28^ (VEP, RRID:SCR_007931), obtaining sequence ontology (SO), gene and predicted severity information. The plugin REVEL^29^ was used to infer the pathogenicity of missense variants, using a 0.5 threshold to identify rare missense variants predicted to be deleterious.

### Rare variant analysis

To assess population structure and identify outlier samples, we considered variants with minor allele frequency (MAF) > 1% and tested their deviation from Hardy–Weinberg equilibrium (HWE) setting a mid p-value^30^ threshold of 1 × 10^−40^ in PLINK^31^ (RRID:SCR_001757, v1.9). To account for linkage disequilibrium (LD), we used the PLINK command “--indep-pairwise” to perform a pruning step, where for each window of 50 variants, each pair showing a squared correlation r^2^ exceeding a 0.2 threshold is greedily pruned from the window. We then calculated and identified the most informative components from the principal component analysis (PCA) and used each of these to remove as outlier every sample that localised more than 8 standard deviations away from the mean.

Only rare variants with minor allele frequency (MAF) < 1% were selected for the analysis and divided into 3 groups based on their predicted severity. Group 1 included only variants showing high damaging impact, according to the VEP annotation (i.e. “Splice acceptor variant”, “Splice donor variant”, “Stop gained”, or “Frameshift variant”; splice variants were considered those affecting intron positions −1/−2 and +1/+2). Group 2 included all variants from Group 1 and missense variants with a “moderate” damaging impact pathogenicity (score >0.5 using REVEL). Group 3 included all variants from Groups 1 and 2, without filtering out missense variants with REVEL.

For each group, variants were then collapsed by genomic region (gene or biological group of genes) and association analyses were performed with a custom implementation of TRAPD^32^ and EPACTS v3.3.0,^33^ which uses an Optimised Sequence Kernel Association Test (SKAT-O),^34^, ^35^, ^36^ and are available at our group GitHub repository.^37^ For each variant group, association p-values were used to compute the genomic inflation factor (λ) and generate quantile–quantile plots. To adjust for genomic inflation, for each group, we used the computed λ to calibrate a minor allele count (MAC) filter that we applied on all genes of that group. The obtained MAC thresholds were 5, 10, and 15 for Group 1, Group 2, and Group 3, respectively, and only genes with false discovery rate (FDR) p ≤ 0.05 were considered as significant. When collapsing variants for multiple genes/pathways, we employed curated biological hallmarks from Gene Set Enrichment Analysis (GSEA, RRID:SCR_003199)^38^ and performed for each group the same association analysis as described above. In this case, we did not apply any MAC filter.

A meta-analysis was conducted to assess the association between rare variants in the *LPO* gene and COVID-19 severity, combining in-house data with publicly available rare variant association statistics from the Regeneron Genetics Center (RGC, https://www.regeneron.com/science/genetics-center). The latter were derived from gene burden testing (including loss-of-function, LoF, and missense variants) in individuals of European ancestry from the Geisinger Health System (GHS) and UK BioBank (RRID:SCR_01281, UKB) cohorts, comparing COVID-19 positive hospitalised cases to COVID-19 negative individuals or those with unknown infection status.

### Bulk RNA-sequencing

Blood samples were collected in 4 mL ethylene-diaminetetraacetic acid-K2 vacuum blood tubes, mixed immediately after the collection by inverting 10 times, and processed less than 8 h after the blood withdrawal. Peripheral blood mononuclear cells (PBMCs) isolation was performed by using Ficoll (Sigma–Aldrich, St. Louis, Missouri, USA) density gradient centrifugation.

Total RNA was isolated from PBMCs using the TriFast reagent (Euroclone; Pero, Milan, Italy). For assessing RNA quantity and quality we used a highly sensitive fluorescence-based quantitation system (Qubit HSRNA; Thermo Fisher Scientific, Waltham, Massachusetts, USA) and the High Sensitivity RNA ScreenTape Assay with an Agilent 4200 TapeStation System (RRID:SCR_018435, Agilent Technologies, Santa Clara, California, USA). Libraries were prepared from 35 to 57 ng of total RNA, using the SMARTer Stranded RNA-Seq Kit (Clontech-Takara; Kusatsu, Shiga, Japan). Quality control was performed using a High Sensitivity DNA D5000 ScreenTape Assay (Agilent Technologies). Libraries were then multiplexed in an equimolar pool and sequenced using an Illumina NextSeq 2000 system (RRID:SCR_023614). An average of 36 million stranded, paired-end 150-nucleotide reads were generated per sample. Quality control for all reads in fastq files was conducted using FastQC (RRID:SCR_014583) v0.11.9 and MultiQC (RRID:SCR_014982) v1.10. Reads shorter than 35 bases and Illumina sequencing adaptors were removed using Trimmomatic (RRID:SCR_011848) v0.36, and the first 3 bases were removed from each read, according to the library manufacturer guideline.

### Differential gene expression analyses

Sequencing reads were mapped on hg38 reference (UCSC) human genome using STAR (RRID:SCR_004463) v2.7.9a (Spliced Transcripts Alignment to a Reference).^39^ To perform novel junction discovery, we run STAR in the 2-pass mode, using the “--twopassMode Basic” option. Quantification of reads mapping on each protein coding gene was obtained using the command “--quantMode GeneCounts”. Subsequent analyses were performed using specific packages available through the R Project for Statistical Computing (RRID:SCR_001905). First, we used “ComBat_seq” from the sva package^40^^,^^41^ (RRID:SCR_012836) to adjust for the latency between hospitalisation and blood collection (either ≤ or >9 days). The DESeq2 (RRID:SCR_015687) R package (v1.34.0)^42^ was used for differential gene expression analysis, dividing the patients in two groups based on disease severity (see Results). Batch effect for sample preparation and patient sex were included in the model. Smoking status was not integrated since only one patient in the dataset was a smoker. We transformed our data using variance stabilising transformation and performed PCA. To account for log2(fold change) inflation of transcript with lower expression, we employed the adaptive shrinkage estimator from the “ashr” R package.^43^ Gene ontology analysis was performed using Metascape^44^ (RRID:SCR_016620) and considering all significant differentially expressed genes, without restricting this analysis with any log2(fold change) threshold but performing the analysis separately for direction of expression.

To better understand the actual contribution of platelets to our RNA-seq signature, canonical platelet and/or megakaryocyte gene expression markers were retrieved from PanglaoDB^45^ (https://panglaodb.se/index.html, accessed 13th November 2025) and compared to the list of our differentially expressed genes (DEGs).

### Splicing analyses

Discovery of novel junction from RNA-seq data was performed using STAR^39^ in the 2-pass mode, using the “--twopassMode Basic” option, and differential AS events between the two groups of patients were detected using Multivariate Analysis of Transcript Splicing (rMATS, RRID:SCR_02348, v4.1.2).^46^ Only junction reads were considered, and we set a maximum exon length of 5000 bases (“--mel” option). splicing events were filtered using an FDR ≤0.05 threshold. Additionally, a minimum absolute difference in inclusion level of 0.05 was applied as a filter for splicing events. The minimum value for both average inclusion and exclusion levels for each group were set to 5 reads.

Information on transcription factors and their target genes in humans have been retrieved from TFLink,^47^ a comprehensive repository of TF-target gene interactions and binding sites downloaded from https://tflink.net/download/ (TFLink_Homo_sapiens_interactions_All_simpleFormat_v1.0.tsv.gz file). By crossing this file with our results, we identified which differentially alternatively spliced genes (DAGs) were also transcription factors, and among their target genes, which ones were also present among our DEGs.

### Statistics

Study design explicitly considered sex among the patient reported variables. The sex distribution of the recruited severe/critical COVID-19 cohorts, both the exome-sequence cohort (N = 215; 151 males and 64 females) and the RNA-seq cohort (N = 59; 41 males and 18 females), reflects the expected over-representation of males among severe cases, as previously reported.^48^

Rare variant association with COVID-19 severity was tested with a cases vs controls design, using EPACTS v3.3.0 (Efficient and Parallelizable Association Container Toolbox)^33^ and SKAT-O (Optimised Sequence Kernel Association Test).^34^, ^35^, ^36^ This implementation allowed us to perform both a burden test, which is more powerful when collapsed variants have an effect on the same direction, and a SKAT test, which accounts for heterogeneous effect size and direction of collapsed variants. The best test was then returned as output. Covariates were taken into account by fitting a null model which uses age, sex, age∗age, age∗sex and the first 10 principal components from the PCA.

Odds ratios (ORs) and their 95% confidence internals (CI) for the significant associations were computed using the “epitools”^49^ R package, with the Haldane-Anscombe correction.

For meta-analysis, p-values from the in-house and RGC datasets (GHS, UKB) were combined using Fisher's method, which aggregates independent p-values by computing the statistic −2∑ln (p_i) and comparing it to a chi-squared distribution with 2 k degrees of freedom to obtain a single meta-analytic p-value. The procedure was implemented via the “sumlog” function in the “metap” R package.

For RNA-seq, significant differentially expressed genes (DEGs) were defined using an adjusted p-value (FDR) ≤ 0.05 and an absolute log2(fold-change) ≥0.5.

Correlation expression analysis between transcription factors and their target was performed for each transcription factor having expression >0 in at least 40% of the samples using R^50^ (RRID:SCR_001905). Spearman correlation was calculated using the “cor.test” function. Only transcription factors with at least two available targets were used for the analysis. This also applied when testing only DEGs as target genes. The mean of the correlations was calculated for (i) only target genes that were DEGs; (ii) only target genes that were not DEGs; and (iii) all target genes. Finally, comparison of medians of mean expression correlations between groups was performed using Wilcoxon rank sum test with the R command “wilcox.test”.

### Role of funders

The funding source had no role in study design; in the collection, analysis, and interpretation of data; in the writing of the report; and in the decision to submit the paper for publication.

## Results

### Rare variants related to oxidative stress drive the association with COVID-19 severity

The hospitalised COVID-19 cohort (215 patients that underwent WES, 151 males and 64 females) had a mean age of 68.9 ± 12.3 years (range 34–94) and displayed a considerable prevalence of cardiometabolic and respiratory comorbidities, including hypertension (54.5%), type 2 diabetes (22.3%), chronic obstructive pulmonary disease (8.1%), and a history of myocardial infarction (18.5%). Additional conditions such as chronic kidney disease (7.1%), arrhythmias (11.8%), and prior or current malignancies were also represented. During hospitalisation, 14.7% of patients died, with 7.6% of deaths directly related to COVID-19. Most individuals required only low-level respiratory support (63.0%), although nearly one-third (28.9%) necessitated advanced interventions. Complications were frequent, including renal failure (10.4%), major bleeding events (including gastrointestinal bleeding and intracranial haemorrhages; 11.4%), deep vein thrombosis (7.6%), and thromboembolism (4.7%). Management strategies predominantly included low-molecular-weight heparin (85.3%) and hydroxychloroquine (70.1%), while corticosteroids were administered in 21.3% of cases and biologics remained uncommon (2.8%) (Table 1).

**Table 1:** Baseline demographic and clinical characteristics of the 215 patients with severe COVID-19 who underwent whole-exome sequencing.

Demographic and anthropometric variables	Age, years	Mean = 68.86; SD = 12.3; Range = 34-94
	Sex, %	Males = 67.7%; Females = 32.3%
	Smoking status, %	Yes = 4.8%; No = 79.6%; NA = 15.6%
	BMI	Mean = 26.9; SD = 5.25; Range = 15.4–45.7
Comorbidities, %	Immunocompromised status	4.3
	Rheumatologic disease	4.3
	Diabetes type 1	0.9
	Diabetes type 2	22.3
	Asthma	2.4
	Chronic obstructive pulmonary disease	8.1
	Liver disease	4.3
	Gallbladder	1.4
	Pancreatic disorders	None
	Chronic kidney disease	7.1
	Congestive heart failure	5.7
	Hypertension	54.5
	Myocardial infarction	18.5
	Peripheral vascular disease	7.1
	Stroke	9.5
	Arrythmias	11.8
	Dementia	5.2
	Neurological or neuropsychiatric disease	8.1
	Lymphoma	1.9 (current)–0.9 (past)
	Leukaemia	2.4 (current)–0.5 (past)
	Malignant solid tumour	5.7 (current)–7.1 (past)
During hospitalisation, %	Death	14.7
	Cause of death	7.6 (due to COVID-19) 8.1 (for other reason)
	Highest level of respiratory support	A = 63.0; B = 8.1; C = 28.9
	Deep vein thrombosis	7.6
	Thrombembolism	4.7
	Myocardial infarction	0.9
	Stroke	4.3
	Renal failure	10.4
	Bleeding	11.4
	Liver failure	3.3
Therapy during hospitalisation	Steroids	21.3
	Biologics	2.8
	Low-molecular-weight heparin	85.3
	Plaquenil	70.1
	Remdesivir	None

A, oxygen (mask, nasal cannula); B, non-invasive ventilation (CPAP, BIPAP, high-flow cannula); C, intubation with or without ECMO; NA, not available; SD, standard deviation. “Biologics” include medications such as abatacept, adalimumab, anakinra, certolizumab pegol, etanercept, golimumab, infliximab, rituximab, tocilizumab, tofacitinib, and upadacitinib.

To characterise the contribution of host genetics to COVID-19 severity, we performed WES on the 215 hospitalised patients and used 1755 previously sequenced Italian individuals as controls.^19^ Sequencing data were harmonised to ensure comparability; we obtained a total of 690,219 rare variants (MAF <1%) in the entire cohort of 1970 individuals.

**Fig. 1:**
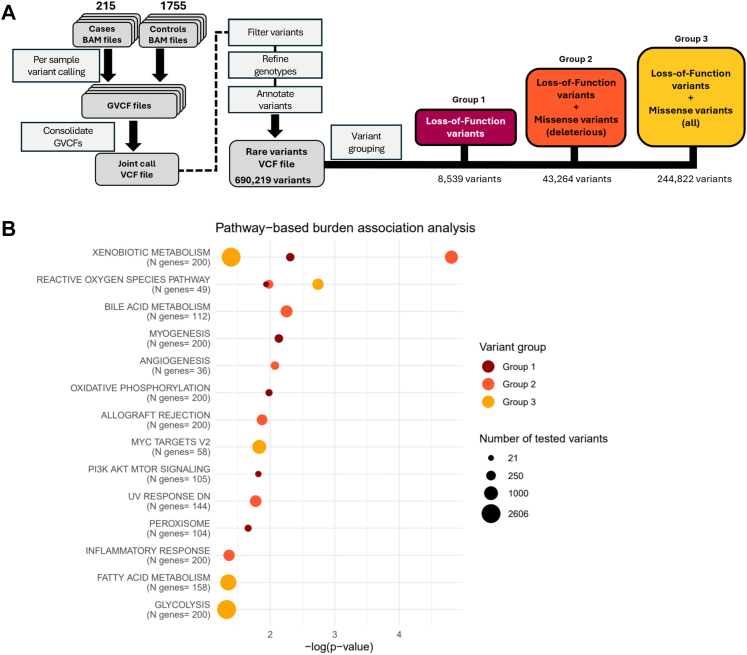
**Rare variant analysis: from gene-based to pathway-based burden test analysis.** (A) Schematic representation of the rare variant analysis workflow starting from WES data. On the left (grey boxes), the GATK germline variant calling pipeline is represented. On the right, aggregation of rare variants into 3 different groups-based on their predicted impact-is shown, from the most stringent category (dark red) to the less stringent one (gold). (B) Burden test analysis by collapsing variants based on biological pathways annotated in the GSEA hallmark gene sets. The bubble plot shows significant combinations of variant group and biological pathways. The x-axis represents the statistical significance of the association, shown as −log_10_ (p-value). The size of each dot corresponds to the number of tested variants in that biological pathway.

Variants were annotated by their predicted severity and categorised into three groups: high-impact LoF variants (Group 1: 8539 variants in 5416 genes); LoF and missense variants predicted to be deleterious (Group 2: 43,264 variants in 11,093 genes); LoF and all missense variants (Group 3: 244,822 variants in 16,061 genes) (see Methods; Fig. 1A). For each group, we performed a gene-based burden test by collapsing all variants located in the same gene and evaluated their association with COVID-19 severity. We identified 3, 11, and 54 significantly associated genes (FDR ≤0.05) (Table 2). For Group 1, the most significant association was with the *MTERF1* gene (FDR = 7.69 × 10^−5^), encoding the mitochondrial transcription termination factor 1, which is involved in the regulation of oxidative phosphorylation.^51^ For both Groups 2 and 3, the gene showing the strongest association was *TDP1* (FDR = 3.23 × 10^−7^ and 1.37 × 10^−6^, respectively), coding for Tyrosyl-DNA Phosphodiesterase 1, which was reported to reduce mitochondrial DNA mutations.^52^ When performing a pathway enrichment analysis on the 64 unique significant genes from all 3 groups, the most significant enriched pathway was “reactive oxygen species metabolic process” (p = 9.12 × 10^−5^; Supplementary Figure S1).

**Table 2:** Gene-collapsed genetic variants significantly associated with COVID-19 severity from burden test analysis.

Genomic location	Gene	MAC	MAC cases	MAC controls	p-value (FDR)	OR	CI (95%)
Group 1							
chr7:91874558-91874558	*MTERF1*	7	3	4	7.69 × 10^−05^	6.59	1.76–24.65
chr1:151579733-151581690	*TUFT1*	6	4	2	3.26 × 10^−02^	13.78	3.28-57.85
chr2:85349504-85349504	*RETSAT*	6	3	3	4.28 × 10^−02^	8.24	2.05–33.08
Group 2							
chr14:89975788-89993402	*TDP1*	11	5	6	3.23 × 10^−07^	7.09	2.37–21.21
chr2:124221793-124914283	*CNTNAP5*	15	7	8	6.64 × 10^−04^	7.39	2.83–19.25
chr20:10639517-10652212	*JAG1*	11	3	8	7.76 × 10^−04^	3.66	1.12–11.93
chr15:28113577-28269306	*HERC2*	20	7	13	1.16 × 10^−03^	4.74	1.98–11.37
chr16:69711200-69726438	*NQO1*	16	7	9	8.03 × 10^−03^	6.65	2.61–16.93
chr11:34449231-34464110	*CAT*	17	4	13	8.03 × 10^−03^	2.94	1.05–8.21
chr11:67423810-67424101	*CARNS1*	18	7	11	8.03 × 10^−03^	5.54	2.25–13.62
chr3:49700639-49713987	*RNF123*	13	2	11	1.31 × 10^−02^	2.05	0.58–7.3
chr6:154089999-154091350	*OPRM1*	15	5	10	3.13 × 10^−02^	4.51	1.66–12.25
chr9:115029453-115090931	*TNC*	29	5	24	3.13 × 10^−02^	1.98	0.81–4.84
chr1:155338371-155480729	*ASH1L*	20	5	15	4.54 × 10^−02^	3.1	1.2–7.95
Group 3							
chr14:89963211-90043081	*TDP1*	24	8	16	1.37 × 10^−06^	4.4	1.95–9.93
chr3:53846596-53865294	*IL1*7RB	32	11	21	4.41 × 10^−04^	4.56	2.24–9.28
chr10:86936789-86957420	*MMRN2*	39	9	30	2.83 × 10^−03^	2.68	1.3–5.5
chr2:47373475-47379863	*EPCAM*	22	7	15	2.83 × 10^−03^	4.15	1.76–9.75
chr13:26214014-26219633	*RNF6*	49	11	38	2.83 × 10^−03^	2.56	1.33–4.93
chr1:24331439-24354423	*GRHL3*	59	16	43	3.75 × 10^−03^	3.25	1.84–5.74
chr12:122473634-122482647	*ZCCHC8*	46	10	36	9.14 × 10^−03^	2.47	1.25–4.88
chr20:53944980-54028936	*BCAS1*	26	9	17	1.35 × 10^−02^	4.63	2.12–10.09
chr16:69710994-69726438	*NQO1*	23	8	15	1.50 × 10^−02^	4.68	2.05–10.65
chr19:17049895-17075409	*HAUS8*	19	4	15	1.50 × 10^−02^	2.57	0.94–7.06
chr2:88526423-88529422	*TEX37*	22	8	14	1.50 × 10^−02^	4.99	2.17–11.47
chr1:26190680-26201445	*CATSPER4*	43	7	36	1.57 × 10^−02^	1.78	0.82–3.85
chr1:152971893-152972074	*SPRR4*	19	9	10	1.58 × 10^−02^	7.59	3.2-17.97
chr17:58247557-58267935	***LPO***	**24**	**6**	**18**	**1.58 × 10^−02^**	**3.05**	**1.27**–**7.29**
chr8:98089174-98090007	*ERICH5*	20	8	12	1.59 × 10^−02^	5.76	2.45–13.56
chr1:99709113-99748642	*FRRS1*	24	5	19	1.78 × 10^−02^	2.47	0.99–6.19
chr11:6794805-6795587	*OR6A2*	25	7	18	2.16 × 10^−02^	3.49	1.52–8.02
chr11:47579280-47584428	*NDUFS3*	24	6	18	2.16 × 10^−02^	3.05	1.27–7.29
chr1:219173906-219211661	*LYPLAL1*	22	4	18	2.20 × 10^−02^	2.17	0.8–5.83
chr7:91873755-91874744	*MTERF1*	33	5	28	2.20 × 10^−02^	1.7	0.7–4.12
chr11:78197956-78213716	*USP35*	94	15	79	2.20 × 10^−02^	1.66	0.96–2.87
chr15:90240933-90241996	*GDPGP1*	53	9	44	2.56 × 10^−02^	1.84	0.92–3.67
chr2:29193257-29920554	*ALK*	64	11	53	2.56 × 10^−02^	1.84	0.98–3.47
chr1:203216939-203229629	*CHIT1*	58	15	43	2.75 × 10^−02^	3.05	1.71–5.45
chr16:27067006-27067224	*C16orf82*	17	4	13	2.75 × 10^−02^	2.94	1.05–8.21
chr19:3179054-3179933	*S1PR4*	51	13	38	2.77 × 10^−02^	3	1.62–5.57
chr15:28111845-28280200	*HERC2*	79	11	68	2.98 × 10^−02^	1.43	0.77–2.67
chr19:18211123-18220897	*PDE4C*	16	3	13	3.60 × 10^−02^	2.35	0.77–7.17
chr10:94441918-94531390	*TBC1D12*	19	5	14	3.60 × 10^−02^	3.3	1.27–8.56
chr14:74286710-74300166	*ABCD4*	79	14	65	3.60 × 10^−02^	1.89	1.07–3.34
chr3:15451673-15521555	*COLQ*	22	4	18	3.83 × 10^−02^	2.17	0.8–5.83
chr3:58508935-58535010	*ACOX2*	53	9	44	3.83 × 10^−02^	1.84	0.92–3.67
chr5:141355235-141511112	*PCDHGA4*	45	9	36	3.83 × 10^−02^	2.24	1.11–4.53
chr7:93192275-93219401	*HEPACAM2*	66	12	54	3.83 × 10^−02^	1.96	1.06–3.62
chr11:65593100-65595592	*KCNK7*	39	10	29	3.83 × 10^−02^	3.05	1.52–6.13
chr1:114097771-114139919	*SYT6*	36	10	26	3.83 × 10^−02^	3.39	1.67–6.89
chr7:151967436-152019710	*GALNTL5*	16	4	12	3.83 × 10^−02^	3.17	1.12–8.94
chr12:101281135-101386059	*UTP20*	206	32	174	3.83 × 10^−02^	1.59	1.08–2.34
chr16:30985729-30988170	*HSD3B7*	34	8	26	3.83 × 10^−02^	2.76	1.29–5.91
chr1:94983111-95072868	*ALG14*	37	7	30	3.86 × 10^−02^	2.13	0.97–4.67
chr17:82058386-82064933	*DUS1L*	38	6	32	3.86 × 10^−02^	1.75	0.77–3.97
chr1:43970233-43972499	*DPH2*	27	4	23	4.01 × 10^−02^	1.71	0.65–4.51
chr16:57759763-57795040	*KIFC3*	35	11	24	4.03 × 10^−02^	4.01	2–8.04
chr14:39175311-39181285	*PNN*	36	10	26	4.08 × 10^−02^	3.39	1.67–6.89
chr10:112155900-112181754	*GPAM*	25	5	20	4.45 × 10^−02^	2.36	0.95–5.87
chr2:65313835-65431978	*SPRED2*	56	13	43	4.45 × 10^−02^	2.66	1.44–4.89
chr13:91448815-92866390	*GPC5*	34	5	29	4.45 × 10^−02^	1.64	0.68–3.97
chr15:77614297-77615612	*LINGO1*	18	3	15	4.45 × 10^−02^	2.05	0.68–6.17
chr11:111688082-111723921	*SIK2*	29	6	23	4.55 × 10^−02^	2.41	1.03–5.62
chr17:76002046-76005331	*CDK3*	24	7	17	4.65 × 10^−02^	3.68	1.59–8.52
chr1:159066229-159073406	*AIM2*	23	7	16	4.72 × 10^−02^	3.9	1.67–9.1
chr5:77030807-77063200	*AGGF1*	25	3	22	4.94 × 10^−02^	1.43	0.49–4.14
chr10:992523-1017204	*GTPBP4*	45	12	33	4.94 × 10^−02^	3.19	1.67–6.1
chr15:47760391-47771771	*SEMA6D*	47	7	40	4.94 × 10^−02^	1.61	0.75–3.45

Abbreviations: SNP: Single Nucleotide Polymorphism; MAC: Minor Allele Count; OR: Odds Ratio; CI (95%): Confidence Intervals at 95% level. Genomic location refers to the gene coordinates on human genome reference GRCh38/hg38. Within Group3 the *LPO* gene, which was further investigated, is shown in bold. N cases = 215; N controls = 1755.

### Pathway-based burden analysis links oxidative stress and interferon pathways to COVID-19 severity

The above-reported results support expanding the rare variant collapsing strategy from single gene to entire pathways, going beyond the traditional approaches in burden test association analysis. We hence tested the cumulative effect of rare variants collapsed on 50 biological pathways from GSEA hallmark gene sets (Supplementary Table S1). For each variant group and for each pathway, we collapsed and tested all the variants present on all genes in that biological pathway. We identified 18 significant variant group–pathway combinations, which included “xenobiotic metabolism”, “reactive oxygen species”, “oxidative phosphorylation”, “PI3K, AKT, MTOR”, “peroxisome”, “inflammatory response”, “fatty acid metabolism”, and “glycolysis” pathways (Fig. 1B). Among the genes included in these pathways, we found *NQO1*, *CAT*, *RETSAT, NDUFS3*, and *HSD3B7,* which were all already found associated with COVID-19 severity from our gene-based burden test analysis (Table 2). Strikingly, the “reactive oxygen species” pathway (including 49 genes) showed a significant association for all 3 tested groups (p = 0.011 calculated on 21 variants, p = 0.010 on 50 variants, and p = 1.82 × 10^−3^ on 486 variants, for Groups 1, 2, and 3, respectively).

Next, we tested the first gene set that was associated with COVID-19 severity in 2020,^53^ focusing on genes involved in regulating type I and III interferon-mediated immunity (nominally, *TLR3, IRF7, IRF9, TICAM1, UNC93B1, TRAF3, TBK1, IRF3, IKBKG, IFNAR1, IFNAR2, STAT1,* and *STAT2*). While we did not find any significant associations when testing these genes individually, the pathway-based analysis evidenced a significant association for Group 1 variants (p = 0.0017).

### Critically severe COVID-19 patients show a prothrombotic transcriptomic profile

**Fig. 2:**
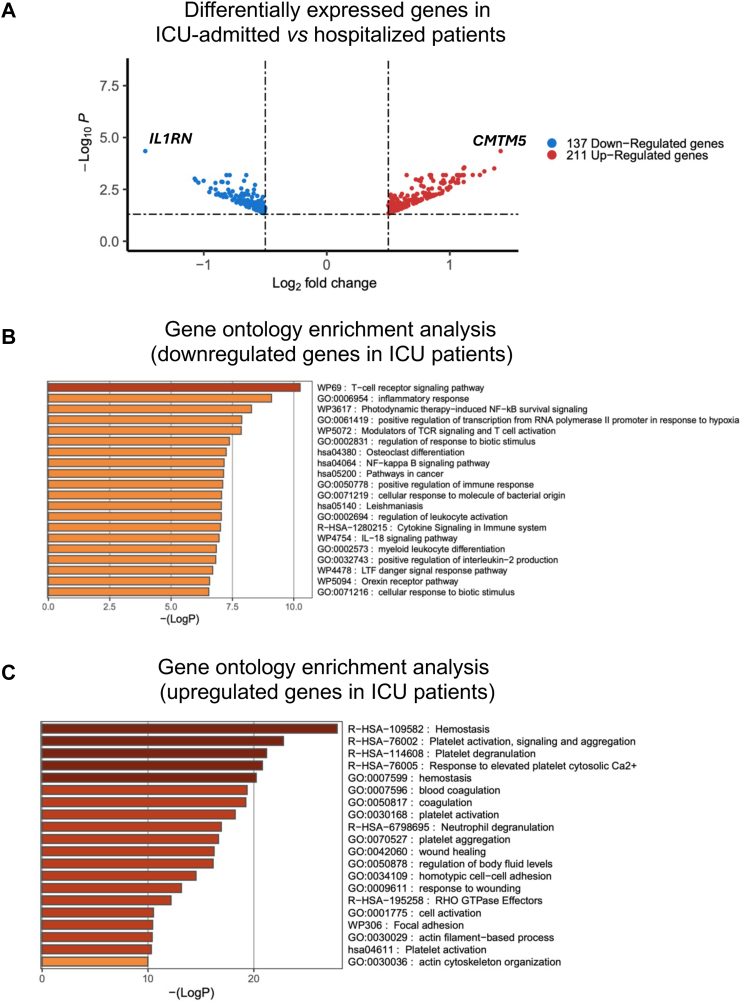
**Comparative transcriptomic analysis between patients admitted to ICU and hospitalised ones.** (A) Volcano plot of DEGs (|log2(fold-change)| ≥0.5 and FDR ≤0.05) in patients stratified for disease severity (ICU-admitted vs hospitalised). Red and blue dots represent upregulated (N = 211) and downregulated (N = 137) genes, respectively. The top up- and downregulated genes are indicated. (B and C) Enrichment analysis of biological processes and pathways downregulated (B) and upregulated (C) in patients stratified for disease severity (ICU-admitted vs hospitalised). Bar plots represent the top 20 enriched pathways, ranked by statistical significance. The x-axis denotes the -log_10_(P) value [Fisher exact test]. The figures were produced using Metascape (https://metascape.org/gp/index.html#/main/step1). N ICU-admitted = 35; N hospitalised = 24 (reference).

To explore possible dysregulated pathways involved in COVID-19 severity, we analysed the transcriptomic profiles of 59 patients (41 males and 18 females) affected by severe COVID-19 symptoms, comparing those who were only hospitalised and required supplemental oxygen support (N = 24, 12 males and 12 females) with those who were also admitted to the ICU and received more intensive respiratory support (N = 35, critically severe, 29 males and 6 females). Details on the ventilatory support, as well as other clinical and demographic features of patients analysed by RNA-seq are reported in Supplementary Table S2. We identified 348 significant DEGs (FDR ≤0.05; absolute log_2_(fold-change) ≥0.5): 211 were upregulated in patients admitted to ICU, whereas 137 were downregulated (Fig. 2A, Supplementary Table S3). The most significant downregulated gene was *IL1RN*, Interleukin 1 Receptor Antagonist (FDR = 4.51 × 10^−5^), while the most significant upregulated was *CMTM5*, CKLF Like MARVEL Transmembrane Domain Containing 5 (FDR = 4.51 × 10^−5^). Table 3 lists the 10 most significant up- and downregulated genes.

**Table 3:** The 10 most significant differentially expressed genes in patients with COVID-19 stratified for severity (ICU-admitted vs hospitalised).

Gene	baseMean	log_2_FoldChange	p-value (FDR)
Top 10 downregulated genes			
*IL1RN*	45.3	−1.48	4.51 × 10^−05^
*ARID4B*	27.42	−0.65	6.48 × 10^−04^
*RASA1*	474.32	−0.82	6.48 × 10^−04^
*EYS*	33.32	−0.79	6.48 × 10^−04^
*CEP83*	39.64	−1.07	9.62 × 10^−04^
*LOC101929512*	33.39	−1.07	1.14 × 10^−03^
*BACH1*	57.34	−0.67	1.22 × 10^−03^
*RNF175*	67.76	−1.00	1.22 × 10^−03^
*TRAPPC13*	43.47	−0.86	1.37 × 10^−03^
*MARCKS*	89.31	−0.85	1.39 × 10^−03^
Top 10 upregulated genes			
*CMTM5*	53.56	1.41	4.51 × 10^−05^
*GSN*	56.66	1.12	2.77 × 10^−04^
*ENOSF1*	140.83	1.11	3.10 × 10^−04^
*SLC24A3*	14.35	1.36	3.10 × 10^−04^
*ITGA2B*	391.46	1.28	4.30 × 10^−04^
*TSPAN33*	27.01	1.19	6.39 × 10^−04^
*LCN2*	35.41	1.24	6.39 × 10^−04^
*VSIG2*	195.79	1.11	6.48 × 10^−04^
*ESAM*	18.07	1.06	6.48 × 10^−04^
*PITPNM2*	44.73	0.85	6.48 × 10^−04^

Abbreviations: ICU: intensive care unit; FDR: false discovery rate; N ICU-admitted = 35; N hospitalised = 24 (reference).

To better understand the transcriptomic differences behind a more severe COVID-19 status, we performed a gene ontology enrichment analysis with Metascape,^44^ including only significant DEGs. When testing downregulated DEGs (N = 137, Fig. 2B), the results showed high enrichment in pathways regulating “inflammatory response” (p = 8.08 × 10^−10^), “NF-kappa B signalling” (p = 6.91 × 10^−8^), “IL-18 signalling” (p = 1.1 × 10^−7^), and “interleukin-2 production” (p = 1.51 × 10^−7^). Gene ontology of upregulated DEGs (N = 211, Fig. 2C) highlighted biological processes such as “haemostasis” (p = 1.34 × 10^−28^), “platelet activation” (p = 1.6 × 10^−23^), and “blood coagulation” (p = 4.34 × 10^−20^).

As platelets lack a nucleus, contain a restricted RNA repertoire, and may retain RNA inherited from their megakaryocyte precursors, we interrogated single-cell RNA sequencing data from PanglaoDB to clarify their contribution to our RNA-seq signature. At the time of access, the resource comprised 305 human samples spanning 74 tissues (1,126,580 cells). From these data, we retrieved 131 canonical platelet markers and 39 megakaryocyte markers, of which 18 were shared. Intersecting these markers with our DEGs revealed 22 platelet and/or megakaryocyte markers among the 211 genes upregulated in patients admitted to ICU, while none of the downregulated genes corresponded to either lineage (Supplementary Table S3). Fourteen of the upregulated markers were platelet-specific, one was megakaryocyte-specific, and seven were shared. These findings support the interpretation that the observed transcriptional signature predominantly reflects platelet biology rather than residual megakaryocyte-derived RNA.

### Genetic and transcriptomic evidence implicates the *LPO* lactoperoxidase gene in severe COVID-19

**Fig. 3:**
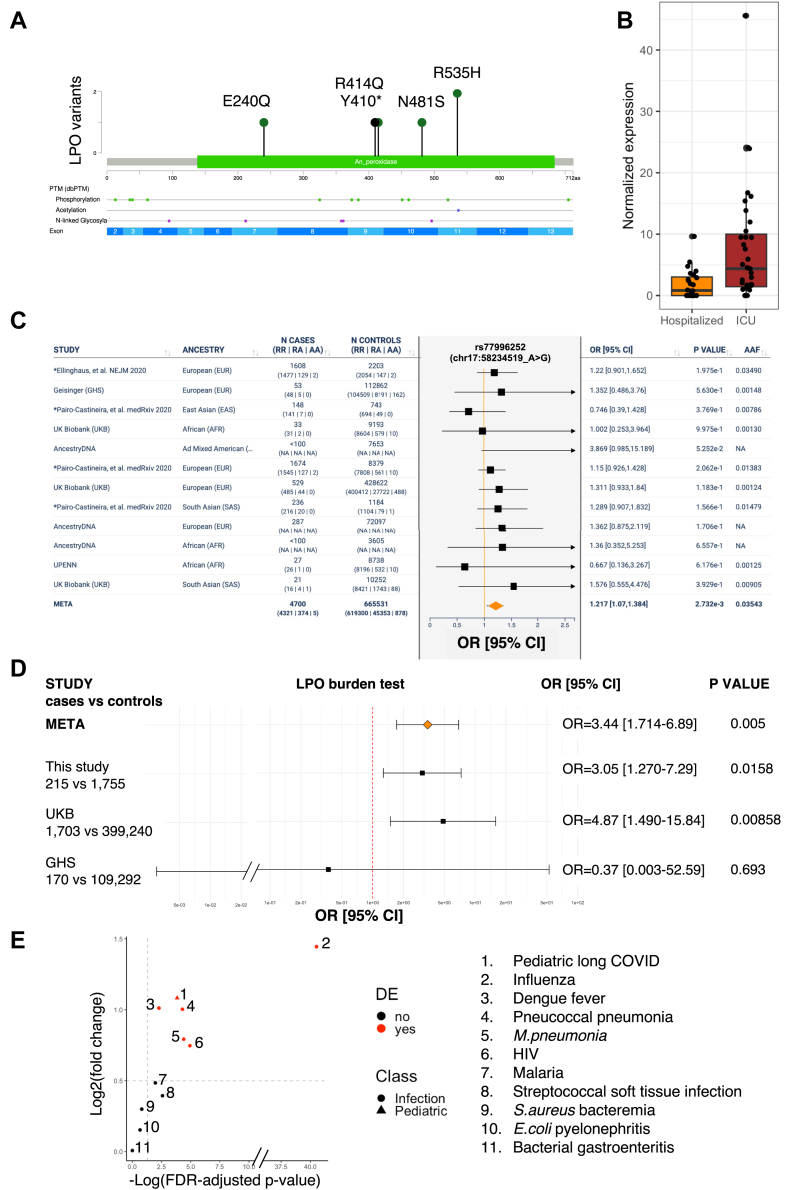
**Genetic and expression evidence of the *LPO* lactoperoxidase gene in COVID-19 severity.** (A) Lollipop diagram of LPO showing the 6 coding variants identified in our COVID-19 patient cohort (green = missense, black = truncating). The diagram was obtained using the MutationMapper tool on the cBioPortal for Cancer Genomics (https://www.cbioportal.org/mutation_mapper). (B) Normalized *LPO* mRNA expression levels in patients admitted to ICU and those hospitalised, as retrieved from RNA-seq data on patients' PBMCs. Each dot represents an individual sample; boxplots show the median, interquartile range, and distribution. (C) Meta-analysis of the association between the common rs77996252 SNP in *LPO* and COVID-19 severity in the RGC database. The Forest plot visualises the individual study ORs, 95% CIs, and the pooled OR. The x-axis is displayed on a log10 scale. Each study's OR, 95% CI, and p-value were displayed alongside the plot. (D) Meta-analysis of the association between rare variants in *LPO* and COVID-19 severity. The Forest plot visualises the individual study ORs, 95% CIs, and the pooled OR. The x-axis is displayed on a log10 scale. Each study's OR, 95% CI, and p-value were displayed alongside the plot. Data include European cohorts (UK Biobank and Geisinger Health System) from the RGC database and our own Italian cohort. (E) Differential LPO expression in disease vs healthy individuals by protein extension assay. Data were retrieved from the Human Protein Atlas. Each dot represents a disease. The x-axis indicates the p-value of significance whereas the y-axis reports the log2(fold-change) in the expression of LPO protein in diseased patients vs healthy controls.

Crossing DEGs with genes showing significant burden of potentially pathogenic variants identified just one gene: *LPO*, which encodes lactoperoxidase, an enzyme that contributes to innate immune response in the airways.^54^^,^^55^ In the burden test, *LPO* showed significant association when using Group 3 variants (p = 1.58 × 10^−2^, OR = 3.05, CI = 1.27–7.29; Table 2). Carriers of *LPO* variants thus had an increased risk of developing severe COVID-19. Fig. 3A shows the location on the LPO protein of the 6 variants (5 missense, one LoF; the p.R535H variant was present in two individuals) identified in patients with COVID-19. In RNA-seq data, *LPO* was significantly upregulated (log2(fold-change) = 0.57, FDR = 0.028) in patients admitted to ICU compared to hospitalised ones (Fig. 3B).

To confirm the genetic association of the *LPO* locus (chr17:58247557-58267935, hg38 coordinates) with COVID-19 in an independent cohort, we retrieved data from the RGC browser (https://rgc-covid19.regeneron.com/home, accessed on 2 April 2025), which includes genome-wide association studies (GWAS) of COVID-19 outcomes across 756,646 individuals (of which >50,000 with COVID-19), derived from four projects: UKB (N = 459,027), AncestryDNA COVID-19 Research Study (N = 163,650), GHS (N = 118,017), and Penn Medicine BioBank (N = 15,952). Association meta-analysis of common variants within *LPO* revealed 59 nominally associated (p < 0.05) single nucleotide polymorphisms (SNPs) with patients with severe COVID-19 compared to individuals with COVID-19 negative/unknown status. The top associated SNP was rs77996252 (p = 0.0027; OR = 1.22, 95% CI = 1.07–1.38; Fig. 3C), located ∼4 kb upstream of the *LPO* transcription start site. Similar results were obtained by exploiting GWAS meta-analyses data from the COVID-19 Host Genetic Initiative (HGI) consortium (https://www.covid19hg.org), as well as from haplotype analysis performed on our Italian COVID-19 GWAS cohort^12^ (Supplementary Materials, Supplementary Figure S2 and Supplementary Table S4).

In line with our results, a significant burden of rare deleterious variants in *LPO* was also found in patients with severe COVID-19 compared to individuals with COVID-19 negative/unknown status in the RGC database. The most significant meta-analysis referred to the pan-ancestry gene burden test including only rare LoF variants (Gene Burden M1; p = 0.0054, OR = 3.53, 95% CI = 1.45–8.57). Among European-only cohorts with data on rare variants (i.e. UKB and GHS), the most significant gene burden was found when including both LoF and missense variants (Gene Burden M2; p = 0.014, OR = 4.24, 95% CI = 1.34–13.36; Supplementary Figure S3). Being the Gene Burden M2 similar to our “Group 3” burden test, we meta-analysed the RGC data with our own results, leading to a significant p = 0.0050, OR = 3.44, 95% CI = 1.71–6.89 (Fig. 3D).

Finally, to better understand whether and how LPO levels are altered in COVID-19 or other disease conditions, we explored publicly available data on LPO protein levels in blood from the Human Protein Atlas (https://www.proteinatlas.org/ENSG00000167419-LPO/blood). Protein profiling results were obtained by Olink proximity extension assay in a Pan-disease cohort. We selected data on all infectious diseases and on paediatric long COVID, and observed that LPO levels were significantly increased (p < 0.05, log2(fold-change) ≥0.5) in all infections characterised by pulmonary symptoms, with higher expression in influenza cases (Fig. 3E).

### Differentially spliced transcription factors impact on the COVID-19 transcriptome

**Fig. 4:**
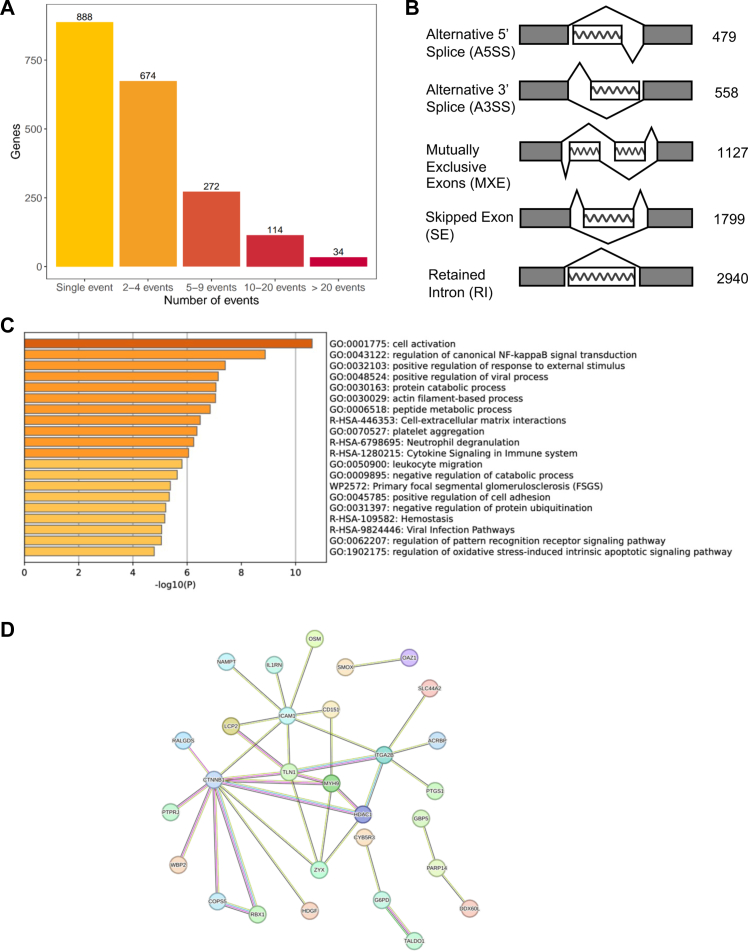
**Analysis of AS in patients with COVID-19 (ICU-admitted compared to hospitalised ones).** (A) Distribution of differential AS events per gene. (B) Number of differential AS events by type. A5SS: alternative 5′ splice site; A3SS: alternative 3′ splice site; MXE: mutually exclusive exons; SE: skipped exon; RI: retained intron. (C) Pathway enrichment analysis performed with Metascape using the top 100 genes harbouring the most significant differential AS events. The x-axis denotes the -log_10_(P) value [Fisher exact test]. (D) Interactome of the 42 genes which display both differential expression and differential AS in patients admitted to ICU compared to hospitalised ones. Only genes that have at least one interactor are shown. Evidence for interaction is based on text mining, experiments, databases, co-expression data, gene fusions, and co-occurrences. Image produced using the STRING tool (https://string-db.org/). N ICU-admitted = 35; N hospitalised = 24 (reference).

To further deepen the analysis of transcriptomic changes associated with COVID-19 severity and gain insight specifically on AS alterations, we leveraged available RNA-seq data from the same 59 patients to perform a differential AS analysis. This allowed us to compare the difference in the isoform ratio of every gene in COVID-19 patients stratified for disease severity (hospitalised and ICU-admitted). We identified 6903 splicing events with significant differences between the two groups (FDR ≤0.05) across 1982 unique genes (Supplementary Table S5). While most of the genes (N = 888) harboured only a single significantly different splicing event, others showed multiple AS events, with the *FGR* gene (FGR Proto-Oncogene, Src Family Tyrosine Kinase) displaying the highest number, namely 53 (Fig. 4A). When considering the type of AS event, alternative 5’ splice site (A5SS) was the least frequent, whereas the most frequent was retained intron (RI) (Fig. 4B). Table 4 lists the top 10 AS events for each type.

**Table 4:** Top 10 genes displaying the most significant differential splicing events (by type) in patients with COVID-19 stratified for severity (ICU-admitted vs hospitalised).

Gene	Chr	Start exon event	End exon event	p-value (FDR)
Alternative 3′ splice site (A3)				
*ACTR3*	chr2	113,934,157	113,934,339	2.2 × 10^−16^
*BNIP3L*	chr8	26,391,242	26,395,294	2.2 × 10^−16^
*FLNA*	chrX	154,349,365	154,352,675	2.2 × 10^−16^
*GNAS*	chr20	58,898,941	58,903,584	2.2 × 10^−16^
*OPTN*	chr10	13,110,273	13,114,491	2.2 × 10^−16^
*GRK6*	chr5	177,433,346	177,433,665	5.3 × 10^−13^
*DOCK8*	chr9	308,189	312,073	9.5 × 10^−13^
*R3HDM4*	chr19	898,776	901,546	1.2 × 10^−11^
*FURIN*	chr15	90,877,526	90,879,284	7.0 × 10^−11^
*WNK1*	chr12	882,942	883,560	7.7 × 10^−11^
Alternative 5′ splice site (A5)				
*FLNA*	chrX	154,352,552	154,352,908	2.2 × 10^−16^
*FURIN*	chr15	90,873,018	90,877,615	2.2 × 10^−16^
*GRK6*	chr5	177,430,067	177,433,410	2.2 × 10^−16^
*GUK1*	chr1	228,145,491	228,148,456	2.2 × 10^−16^
*MPP1*	chrX	154,784,027	154,785,952	2.2 × 10^−16^
*OPTN*	chr10	13,108,953	13,110,476	2.2 × 10^−16^
*PPIA*	chr7	44,797,325	44,799,874	2.2 × 10^−16^
*SEPT2*	chr2	241,335,134	241,335,351	2.2 × 10^−16^
*UNC119*	chr17	28,547,998	28,548,607	2.2 × 10^−16^
*CSDE1*	chr1	114,730,257	114,732,868	3.11 × 10^−13^
Retained intron (RI)				
*DCAF11*	chr14	24,115,170	24,118,155	2.2 × 10^−16^
*EHBP1L1*	chr11	65,584,240	65,584,530	2.2 × 10^−16^
*EIF1*	chr17	41,689,748	41,690,189	2.2 × 10^−16^
*EPB41*	chr1	29,033,092	29,035,921	2.2 × 10^−16^
*FLNA*	chrX	154,352,552	154,352,908	2.2 × 10^−16^
*FLOT1*	chr6	30,738,156	30,742,676	2.2 × 10^−16^
*GPS2*	chr17	7,313,207	7,315,119	2.2 × 10^−16^
*HPSE*	chr4	83,302,149	83,306,713	2.2 × 10^−16^
*IRAK1*	chrX	154,014,041	154,016,093	2.2 × 10^−16^
*MARCH6*	chr5	10,397,292	10,400,840	2.2 × 10^−16^
Mutually Exclusive Exons (MXE)				
*ARRB2*	chr17	4,716,411	4,716,608	2.2 × 10^−16^
*BNIP3L*	chr8	26,391,242	26,391,426	2.2 × 10^−16^
*DOCK8*	chr9	308,189	308,289	2.2 × 10^−16^
*FLNA*	chrX	154,351,883	154,352,021	2.2 × 10^−16^
*MPP1*	chrX	154,784,027	154,784,108	2.2 × 10^−16^
*OPTN*	chr10	13,104,776	13,109,288	2.2 × 10^−16^
*RAB10*	chr2	26,104,923	26,109,906	2.2 × 10^−16^
*RNF10*	chr12	120,565,427	120,565,523	2.2 × 10^−16^
*RNF213*	chr17	80,381,285	80,383,928	2.2 × 10^−16^
*SMARCC2*	chr12	56,170,143	56,170,208	2.2 × 10^−16^
Skipped Exon (SE)				
*ADIPOR1*	chr1	202,954,240	202,954,307	2.2 × 10^−16^
*ARRB2*	chr17	4,718,260	4,718,351	2.2 × 10^−16^
*DOCK8*	chr9	308,189	311,300	2.2 × 10^−16^
*FLNA*	chrX	154,352,554	154,352,675	2.2 × 10^−16^
*GRK6*	chr5	177,433,315	177,433,410	2.2 × 10^−16^
*GUK1*	chr1	228,148,370	228,148,456	2.2 × 10^−16^
*HIPK1*	chr1	113,952,767	113,954,770	2.2 × 10^−16^
*HMGN1*	chr21	39,345,829	39,345,958	2.2 × 10^−16^
*ITGB1*	chr10	32,911,887	32,912,124	2.2 × 10^−16^
*KPNA6*	chr1	32,139,178	32,139,864	2.2 × 10^−16^

Abbreviations: Chr: chromosome; FDR: false discovery rate; Genomic location refers to the gene coordinates on human genome reference GRCh38/hg38. N ICU-admitted = 35; N hospitalised = 24 (reference).

When testing all 1982 unique significant differentially alternatively-spliced genes (DAGs) through a gene ontology analysis, we found “metabolism of RNA” (p = 1.24 × 10^−60^) and “Cytokine Signalling in Immune system” (p = 1.37 × 10^−53^) among the most enriched pathways (Supplementary Figure S4). This suggested that some genes harbouring AS events were also the ones involved in the metabolism of RNA itself. Interestingly, when performing the same analysis using only the top 100 genes containing the most significant AS events (independently from the event type), we found “regulation of NF-kappa B signal” (p = 1.32 × 10^−9^), “regulation of viral process” (p = 7.12 × 10^−8^), “platelet aggregation” (p = 4.35 × 10^−7^), “neutrophil degranulation” (p = 5.75 × 10^−7^), “cytokine signalling” (p = 8.85 × 10^−7^), and “haemostasis” (p = 6.67 × 10^−6^) (Fig. 4C). These pathways overlapped with those identified from our DEG analysis, further supporting the role of these molecular mechanisms in influencing the COVID-19 phenotype. These results were consistent also when performing a gene ontology analysis for each type of AS event separately (not shown). Starting from this observation, we crossed the lists of significant genes derived from the AS (N = 1982) and DEG (N = 348) analyses and found 42 shared genes, most of which are interconnected in a unique network according to STRING analysis (Protein–protein interaction -PPI- enrichment p-value: p = 0.00045; Fig. 4D). Among them, we found *IL1RN*, the most significant downregulated gene, and *ITGA2B*, the 5th most significant upregulated gene.

**Fig. 5:**
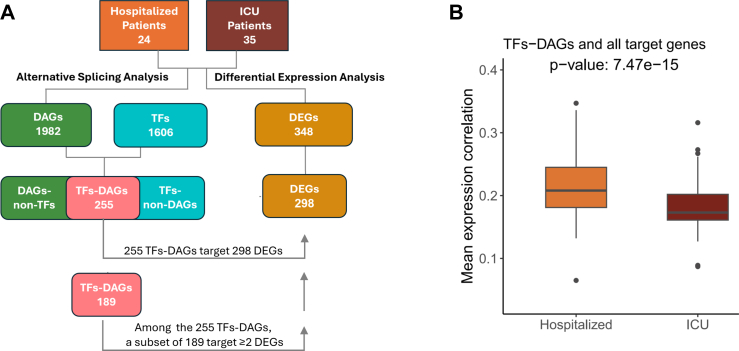
**Correlation between expression levels of differentially-spliced transcription factors (TF-DAGs) and their target genes.** (A) Schematic representation of the strategy used to identify: i) genes harbouring differential splicing events (DAGs), ii) transcription factors (TFs, retrieved from the TFlink database), iii) differentially-spliced TFs (TF-DAGs), iv) differentially expressed genes (DEGs), and v) subset of TF-DAGs targeting at least 2 DEGs (N = 189). (B) Mean expression correlation of TF-DAGs and their target genes in patients with COVID-19 (hospitalised and ICU-admitted). Boxplots show the mean Spearman correlation between TFs–DAGs (N = 197) expression and the expression of their target genes in patients with COVID-19: hospitalized (orange) and ICU-admitted (dark red). The Wilcoxon rank sum test was used to assess the statistical significance.

To further investigate the relationship between DEGs and DAGs, we gathered transcription factor (TF)-target gene interaction information from the TFlink database.^47^ We hypothesised that differential splicing events could alter protein isoforms, potentially impacting their function, such as the ability of TFs to regulate target genes (e.g. those observed in our DGE analysis). Among the 1982 identified DAGs, 255 were annotated as TFs. When we searched for an overlap between their target genes and the DEGs resulting from our bulk RNA-seq analysis, we found that most of our DEGs, nominally 298 out of 348 (85%), are targets of TFs harbouring significant splicing events (TFs-DAGs). In line, we found that 189 out of 255 TFs-DAGs (74%) had at least two DEGs among their targets (Fig. 5A). We hence tested the hypothesis that differential AS events in a TF can reflect in a different correlation between the TF and its target genes expression in patients admitted to ICU vs hospitalised ones. Our results showed that differentially spliced TFs have a reduced correlation between their own expression and the expression levels of their target genes in patients with more severe COVID-19 symptoms. We observed this phenomenon both when testing only DE target genes (N = 189; p = 3.48 × 10^−2^; data not shown) and all target genes (N = 197; p = 7.47 × 10^−15^; Fig. 5B; and Supplementary Table S6).

## Discussion

Here, we leveraged different bioinformatics methods and multiple omics layers to investigate predisposition to COVID-19 severity in the Italian population. We adopted two complementary approaches: on one hand, an exome-wide case–control design for investigating rare germline variant contribution to severe disease susceptibility; on the other hand, a patient-only transcriptomic analysis to highlight signatures associated with a critically ill phenotype (e.g. need for admission to ICU and mechanical ventilation, such as intubation or ECMO).

Gene-level association analysis revealed *MTERF1*, *TUFT1* (encoding Tuftelin1), and *RETSAT* (encoding retinol saturase) as the most significantly associated genes, all enriched for LoFs in patients with COVID-19 (Table 2). These genes are involved in the regulation of oxidative stress and ROS production: *MTERF1* in oxidative phosphorylation,^51^ *TUFT*1 in PI3K/AKT signalling and autophagy under hypoxia via HIF-1α,^56^, ^57^, ^58^, ^59^ and *RETSAT* in ROS generation and cellular oxidative response.^60^ In addition, we further dissected the impact of rare variants expanding the burden analysis from single gene to biological pathways, which can increase the statistical power to detect biologically relevant associations.^61^ Consistently, we found enrichment in processes related to oxidative stress, including “reactive oxygen species”, “oxidative phosphorylation”, and “PI3K-AKT-mTOR” (Fig. 1B). Interestingly, this approach allowed us to replicate previous findings from Zhang et al. (2020), through a “supervised” pathway analysis on their Type-I-interferon gene signature.^53^

Transcriptomic analysis comparing patients with less severe disease (hospitalised, serving as reference) and the critically ill ones (ICU-admitted) revealed distinct molecular signatures reflective of disease progression.^8^ Importantly, these findings are unlikely to be a consequence of comorbidities, as the percentage of patients with pre-existing conditions is similar in the two groups: 13 out of 24 (54%) in the hospitalised group and 22 out of 35 (63%) in the ICU group (not statistically significant, chi-square = 0.45, p-value = 0.51). Specifically, patients with less severe disease exhibited a predominant proinflammatory profile (Fig. 2B), while individuals admitted to ICU showed marked upregulation of prothrombotic genes (Fig. 2C). Among those, *CMTM5*, *ITGA2B*, and *SLC24A3* are implicated in platelet function and cardiovascular complications,^62^ whereas *ENOSF1* has been previously linked to hypertension.^63^ These results are corroborated by pathway enrichment analysis of the genes harbouring the most significantly altered splicing events, which again highlighted pathways linked to inflammation and coagulation/haemostasis (Fig. 4C). Notably, among the top AS genes we found the proinflammatory *IL1RN* (the most significant upregulated in hospitalised-only individuals), and the prothrombotic *ITGA2B* (the 5th top upregulated gene in ICU-admitted cases) (Table 3). *IL1RN* encodes IL-1Ra, which acts as a receptor antagonist and has potent anti-inflammatory activity.^64^ Interestingly, IL-1Ra intracellular isoforms, presumably generated by AS, have been identified.^65^ The actual significance of expression of secreted vs intracellular IL-1Ra remains to be investigated, but the results reported here suggest that AS of *IL1RN*, possibly affecting the balance between secreted and intracellular IL-1Ra, may have an important impact in systemic inflammation.

Viral infection can typically induce AS events of host RNAs, and abnormal alternative RNA splicing has been observed in patients with COVID-19.^66^ Indeed, we noticed that more than 85% of our DEGs were targets of TFs that also harboured differentially spliced events (nominally 298/348; Fig. 5A). In an attempt to evaluate the impact of splicing events on the transcriptome, we computed the correlation between the expression levels of TFs and their predicted target genes. Our results suggest that the presence of differential splicing events could impact on the activity of afflicted TFs and contribute to the transcriptomic differences between patients with COVID-19 stratified for disease severity (hospitalised and ICU-admitted, Fig. 5B).

Finally, the integration of the two omics approaches (exome- and transcriptome-based) pinpointed a single gene, *LPO*, which was significantly enriched in damaging rare variants in patients with COVID-19, and was also significantly upregulated in ICU-admitted individuals (Fig. 3A and B). LPO is physiologically overexpressed (at protein and/or mRNA level) as a defence mechanism in response to airway infections (both bacterial and viral)^54^^,^^55^; the degree of overexpression may depend on the specific pathogen (e.g. patients with influenza had the highest LPO upregulation among tested infectious diseases; Fig. 3E) or on disease severity (e.g. patients admitted to ICU had higher levels of *LPO* than hospitalised ones; Fig. 3B). Hence, *LPO* haploinsufficiency -due to possibly inactivating mutations-might predispose to develop COVID-19 by impairing oxidative stress responses and the ability of the organism of neutralising harmful ROS, particularly H_2_O_2_.^67^ Strikingly, a proposed factor for Japan low COVID-19 death rate is the sustained function of the LPO system. This protective effect is linked to environmental factors, specifically a diet high in iodine, which is essential for the LPO system antimicrobial activity.^68^ Besides playing an important role in keeping under control oxidative stress during infections, impairment of the LPO system may provide a functional link also with the progression of COVID-19 to the final stage of MOF. Indeed, *LPO*-knockout mice exhibited multi-organ inflammation and lesions, involving the cardiovascular, respiratory, digestive (e.g. liver and intestine) and excretory systems.^67^

Our study presents methodological and conceptual strengths that enhance the robustness of the findings. By integrating WES, bulk transcriptomics, and AS analyses generated from the same clinically well-characterised cohort, we provide a multilayered view of the biological determinants of COVID-19 severity that is rarely achieved in genomic studies of infectious diseases. The use of ancestry-homogeneous Italian cases and controls minimises technical and population-related biases in variant discovery. A distinctive strength of our work is the implementation of an in-house pathway-level rare variant collapsing approach, which extends traditional gene-centric burden testing to biologically coherent gene sets, thus allowing the detection of a significant cumulative mutational load across pathways implicated in oxidative stress, metabolic regulation, and interferon signalling. The association involving *LPO* is strengthened by replication in large external datasets (e.g. RGC), supporting the generalizability of our findings. Finally, the comprehensive dissection of AS alterations, particularly in transcription factors whose regulatory relationships are disrupted in critically ill patients, adds mechanistic insights by revealing layers of post-transcriptional dysregulation that contribute to the severe COVID-19 phenotype.

However, our study suffers from some limitations that should be acknowledged: 1) a relatively small sample size in both exome-wide and RNA-seq analyses, which could limit its statistical power; 2) the use of PBMCs that might not completely capture the effects of SARS-CoV-2 infection on the host transcriptome. Despite these limitations, we provide substantial evidence into COVID-19 pathophysiology in the Italian population, laying a foundation for future research to better identify and protect individuals at greater risk.

In conclusion, we found the dysregulation of oxidative phosphorylation and overproduction of ROS to be among the major drivers of COVID-19 severity, with *LPO* emerging as player in disease susceptibility and progression. In the future, it will be interesting to further investigate the role of *LPO* and the impact of AS dysregulation in COVID-19.

## Contributors

Conceptualization: R.A.; Data curation: C.C., F.T., G.S.; Formal analysis: C.C., E.M.P., F.T., G.S., R.A; Funding acquisition: R.A., A.M.; Investigation: V.R., G.C.; Project administration: R.A.; Resources: R.A., A.M.; Software: C.C., E.M.P.; Supervision: R.A.; Visualization: C.C., G.S.; Writing—original draft: C.C; Writing—review & editing: R.A., C.C., G.S., A.M. C.C, G.S, F.T and R.A accessed and verified the underlying data.

All authors read and approved the final version of the manuscript.

## Data sharing statement

All individual participant data underlying the results reported in this article, after de-identification, will be made available immediately upon publication. Clinical and demographic variables used in this study are provided within the main manuscript and the Supplementary Material and are fully accessible upon publication.

WES data for healthy controls are deposited in the NIH dbGAP repository under accessions phs000279 and phs000806. These datasets are available through controlled access, following dbGaP standard procedures, which require submission and approval of a data access request and a signed data use agreement.

WES summary statistics for COVID-19 cases are deposited in ZENODO under accession number https://doi.org/10.5281/zenodo.16326414. The record is publicly accessible, but the access to files is restricted and available upon request to the corresponding author.

Raw and processed RNA-seq data have been deposited in ArrayExpress at EMBL-EBI under accession number E-MTAB-15584 (https://www.ebi.ac.uk/biostudies/arrayexpress/studies/E-MTAB-15584) and will be made publicly available without restriction immediately upon publication.

## Declaration of interests

No conflicts of interest to declare.
