# Supplementary material for: Multi-omics identifies oxidative stress, prothrombotic pathways, and lactoperoxidase variants as key factors in COVID-19 severity
Source: eBioMedicine. 2026 Jan 6;123:106111. doi: 10.1016/j.ebiom.2025.106111 (PMC12809084; doi:10.1016/j.ebiom.2025.106111)
Supplement: Supplementary Materials [file mmc1.pdf]

Supplementary materials to the manuscript: "Multi-omics identifies oxidative stress, prothrombotic pathways, and lactoperoxidase variants as key factors in COVID-19 severity

By Claudio Cappadona, Valeria Rimoldi, Francesca Tettamanzi, Giulia Cardamone, Alberto Mantovani, Giulia Soldà, Elvezia Maria Paraboschi, Rosanna Asselta

List of content:

Supplementary methods

Supplementary results

Supplementary figure 1. Pathway enrichment analysis of the 64 unique significant genes identified through gene-based burden test association analysis with severe COVID-19.

Supplementary figure 2. Analysis of common SNPs in the LPO locus: genetic association with severe COVID-19 and eQTL effects in whole blood.

Supplementary figure 3. Meta-analysis of the association between rare variants in the LPO gene and COVID-19 severity in European cohorts.

Supplementary figure 4: Pathway enrichment analysis of the 1,982 unique genes harboring significantly dysregulated alternative splicing events.

Supplementary table 1. Pathway-based burden test association analysis.

Supplementary table 2. Demographic and clinical features of 59 patients selected for RNA-seq analysis. (available as separate Excel file)

Supplementary table 3. Significant differentially expressed genes.

Supplementary table 4. Haplotype association analysis in the *LPO* locus.

Supplementary table 5. Significant splicing events. (available as separate Excel file)

Supplementary table 6. Mean expression correlation of differentially alternatively spliced (DAG) transcription factors (TF) and their target genes. (available as separate Excel file)

Supplementary references

SUPPLEMENTARY METHODS

Common-variant architecture of the *LPO* region

a) Analyses based on publicly available data

Summary statistics for severe COVID-19 were obtained from the COVID-19 Host Genetics Initiative (HGI; https://www.covid19hg.org/), using the *A2_ALL_eur* dataset comprising 5,101 very severe respiratory confirmed COVID-19 cases and 1,383,241 population controls of European ancestry. The analysis focused on the *LPO* locus, defined as chr17:58.24-58.27 Mb (GRCh38), corresponding to the genomic coordinates of the *LPO* gene extended by 50 kb upstream and downstream. All single nucleotide polymorphisms (SNPs) within this interval were extracted and examined using the P values reported by HGI. Variants with $P<0.05$ were considered nominally significant. The strongest signals were identified by ranking SNPs according to their association statistics, highlighting those with $P<5\times10^{-3}$. The regional association plot was generated in R (version 4.2.3) using the ggplot2 package.

To explore potential regulatory effects, the top associated variants were annotated using the GTEx Portal (v8; https://gtexportal.org/home/), and those reported as significantly associated with *LPO* expression in whole blood were classified as *LPO* eQTLs.

The LD structure of the *LPO* region was reconstructed using the 21 SNPs nominally associated in HGI, and the LDmatrix tool within the LDlink suite (https://ldlink.nih.gov), based on the 1000Genomes Project data (GRCh38) from European populations (excluding Finnish individuals). Pairwise LD (r^2) values were used to define the correlation structure across the region.

b) Haplotype reconstruction and association testing in the Italian dataset

Haplotype reconstruction, frequency estimation, and haplotype association analyses were performed with PLINK v1.07 [Purcell et al., 2007] on our Italian cohort, comprising 527 severe cases and 3215 population-based controls. In cases [median age of 61±8.56 (range: 18-90); 70.4% males], severe COVID-19 was defined as hospitalization with respiratory failure and a confirmed SARS-CoV-2 viral RNA PCR test from nasopharyngeal swabs. Patients were recruited in the period March-May 2020 from intensive care units and general wards at three hospitals in Lombardy, i.e., the Humanitas Clinical and Research Center, IRCCS, in Rozzano, Italy (180 patients); the San Gerardo Hospital, in Monza, Italy (320 patients); and the Humanitas Gavazzeni hospital, in Bergamo, Italy (27 patients). Controls were from the general Italian population with unknown COVID-19 status [median age of 68±12.35 (range: 25-99); 61.9% males]. Approvals for the project were obtained from the relevant ethics committees (for Humanitas hospitals, reference number 316/20; for the University of Milano-Bicocca School of Medicine, San Gerardo Hospital, reference number 84/2020). The requirement for informed consent was waived. Details on DNA extraction, array genotyping, imputation, and quality checks are reported in [Degenhardt et al., 2022]. The final set of analyzed variants comprised 19 SNPs, distributed in the *LPO* region, surrounding the rs57397900 polymorphism. Age, sex, age*age, sex*age, and the first 10 principal components of ancestry were introduced in the model as covariates.

The dataset for 332 COVID-19 patients is publicly available at the European Bioinformatics Institute (www.ebi.ac.uk/gwas) under accession numbers GCST90000255 and GCST90000256 [Severe Covid-19 GWAS Group, 2020]; the dataset for 1668 healthy individuals of the general population is deposited in the Genotypes and Phenotypes database (https://www.ncbi.nlm.nih.gov/gap/) under the phs000294.v1.p1 accession code [Myocardial Infarction Genetics Consortium, 2009]. Data for the remaining patients and controls are available from the corresponding author upon reasonable request, until their deposition in a public repository (pending the acceptance of an unrelated manuscript).

SUPPLEMENTARY RESULTS

To further support the potential involvement of the *LPO* locus to the genetic susceptibility to severe COVID-19, we explored the contribution also of common genetic variants located in the region, by analyzing summary statistics from the COVID-19 HGI consortium (comprising 5,101 very severe respiratory confirmed COVID-19 cases and 1,383,241 population controls). Within the regional association interval encompassing the *LPO* gene: i) 21 of the 225 examined SNPs (9.3%) displayed nominal evidence of association (P<0.05); ii) four of them were already observed among the 59 nominally associated in the Regeneron dataset (see the Results in the main text); and iii) five variants showed the strongest signals ($P<5\times10^{-3}$). The lead variant was rs57397900 (chr17:58222906:G:A; $P=1.26\times10^{-3}$; OR=1.16; 95%CI=1.05-1.26, referring to the reference allele), located in the *LPO* promoter region (Supplementary figure 2A). Notably, three of these five top variants are reported as *LPO* eQTLs in whole blood according to the GTEx portal (Supplementary figure 2B).

Since HGI data are limited to summary statistics, they do not permit more sophisticated analyses such as haplotype reconstruction. To overcome this, we first reconstructed the LD structure of the region using the 21 SNPs nominally associated in HGI, combined with 1000 Genomes Project data (hg38) from European populations (excluding Finnish individuals). Interestingly, this analysis revealed that the top-associated SNP does not reside within any discernible haplotype block (Supplementary figure 2C). Using this LD-informed framework, we then conducted haplotype analyses in our Italian cohort, which included 527 severe COVID-19 cases and 3,215 population-based controls. Given the absence of a defined LD block around the lead SNP, we implemented a sliding-window haplotype association approach, using windows that always included the top-associated rs57397900 SNP. To mitigate the issue of excessive multiple testing while still capturing local haplotypic structure, we selected a window size of 10 SNPs. This strategy allowed us to evaluate regional effects without generating an inflated number of rare haplotypes. In the omnibus analyses, 5 out of the 10 tested windows showed nominal significance (P<0.05), with signals gradually shifting from the promoter-proximal region toward the coding portion of the *LPO* gene (Supplementary figure 2D). The strongest omnibus association (P=0.015) highlighted the presence of two haplotypes exhibiting significantly different frequencies between cases and controls, underscoring a potentially meaningful local haplotypic architecture that is not captured by the lead SNP alone (Supplementary Table 4).

Collectively, our results suggest that while the lead SNP rs57397900 provides an initial signal, the genetic susceptibility at the *LPO* locus may be better explained by local haplotypic architecture, which encompasses variants linked to *LPO* expression (eQTLs).

Supplementary figure 1: Pathway enrichment analysis of the 64 unique significant genes identified through gene-based burden test association analysis with severe COVID-19.

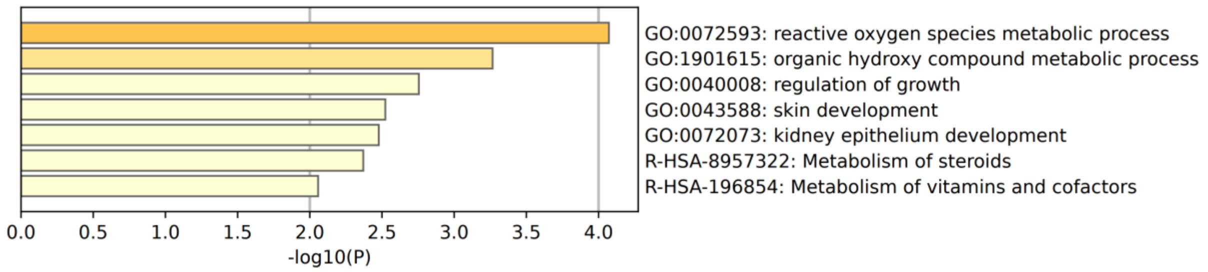

Enrichment analysis of biological processes and pathways. The bar plot represents the top enriched Gene Ontology (GO) biological processes and Reactome pathways, ranked by statistical significance. The most significantly enriched term, *GO:0072593 - reactive oxygen species metabolic process*, is highlighted in a darker shade. The x-axis denotes the $-\log_{10}(P)$ value [Fisher's exact test]. The figure was produced using Metascape (https://metascape.org/gp/index.html#/main/step1).

Supplementary figure 2. Analysis of common SNPs in the *LPO* locus: genetic association with severe COVID-19 and eQTL effects in whole blood.

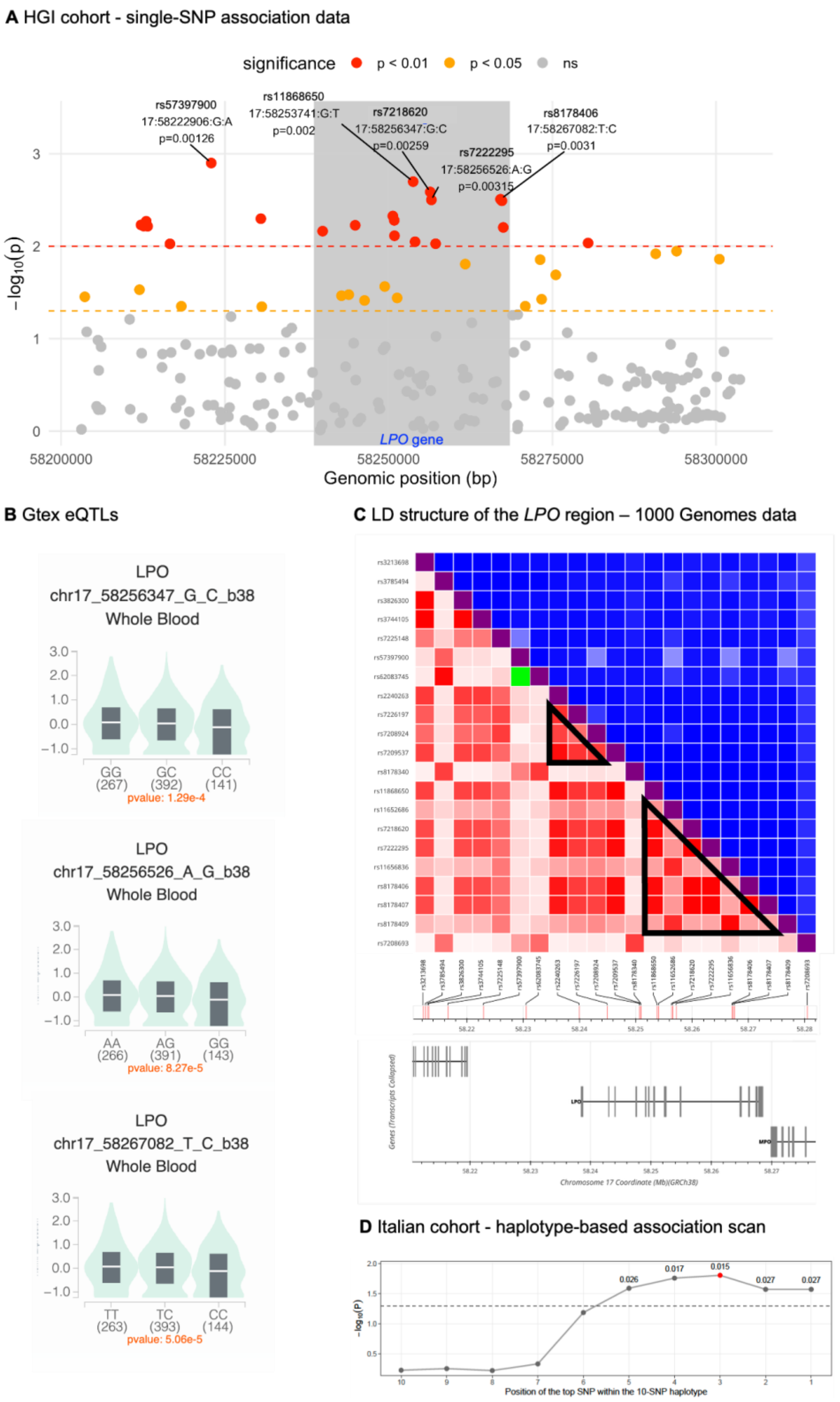

A) Regional association plot depicting the relationship between SNPs and COVID-19 susceptibility within the *LPO* genomic region (±50 kb; 225 SNPs in total; P<0.00022) [Bonferroni-corrected significance threshold]. SNPs are plotted according to their genomic position (x-axis) and $-\log_{10}$(P-value) (y-axis), based on summary statistics from HGI (European ancestry; very severe respiratory, laboratory-confirmed COVID-19 cases versus the general

population). The shaded grey area denotes the *LPO* gene region. The five most significant variants are annotated with their rsIDs and corresponding p-values. Dashed horizontal lines indicate nominal significance thresholds (P=0.05 and P= 0.01).

B) Violin plots illustrating the association between genotype and *LPO* expression levels in whole blood for three top-associated variants. For each SNP, normalized expression values are shown across the three genotype groups, with sample sizes indicated in parentheses. The median and interquartile ranges are represented by overlaid boxplots. Corresponding P values for the genotype-expression association are reported below each panel.

C) Top panel: Pairwise LD between 21 SNPs nominally associated with severe COVID-19 in the HGI dataset, calculated using European populations from the 1000 Genomes Project (hg38, non-Finnish). LD is represented as r^2 values, with a color gradient from blue (low LD, $r^2 \approx 0$) to red (high LD, $r^2 \approx 1$). Two major LD blocks are highlighted by black triangles, while the lead variant rs57397900 (chr17:58222906:G:A), not residing within any LD haplotype block, is marked by a green square. SNP positions (rsIDs) are indicated along the x- and y-axes.

Bottom panel: Gene annotation of the *LPO* locus (chr17:58.24-58.27 Mb, GRCh38) showing the positions of *LPO* and the adjacent *MPO* gene. Vertical lines represent the location of exons within each gene

D) Sliding-window haplotype association scan for the *LPO* locus. The plot shows the –log10(P) values from omnibus haplotype tests across ten overlapping 10-SNP windows, all containing the top-associated SNP (rs57397900, chr17:58222906:G:A). Each point represents one window, with the window showing the lowest P-value highlighted in red. Horizontal dashed line indicates the nominal significance threshold (P=0.05). The X-axis shows the position of the top SNP within each haplotype window, numbered from 10 to 1 for visualization of the sliding-window direction.

Supplementary figure 3: Meta-analysis of the association between rare variants in the *LPO* gene and COVID-19 severity in European cohorts.

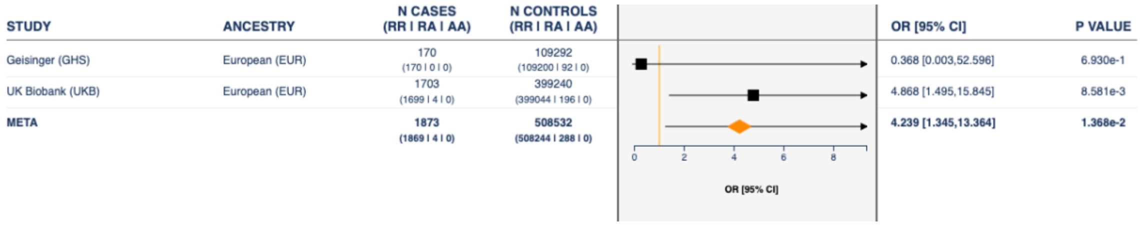

Data have been retrieved from the Regeneron Genetic Center (RGC) results browser (https://rgc-covid19.regeneron.com/home, last accessed on April 2, 2025), which include rare variants association results on European populations (UK Biobank and Geisinger Health.

The Forest plot visualizes the individual study ORs, 95% CIs, and the pooled OR with 95% CI. Each study's OR, 95% CI, and P-value were displayed alongside the plot.

Supplementary figure 4: Pathway enrichment analysis of the 1,982 unique genes harboring significantly dysregulated alternative splicing events.

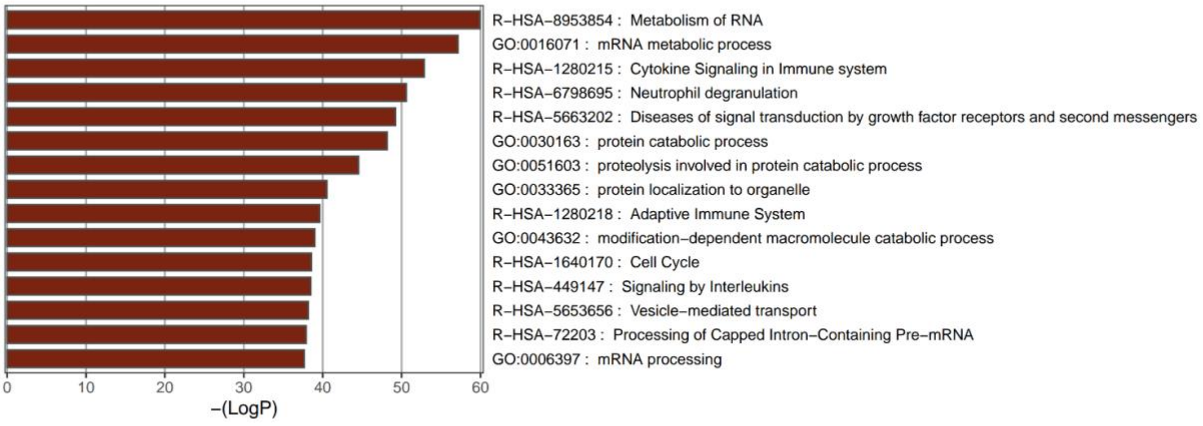

Enrichment analysis of biological processes and pathways, performed on 1,982 unique genes harboring significantly dysregulated alternative splicing events. Bar plots represent the top enriched Gene Ontology (GO) biological processes and Reactome pathways (R) pathways, ranked by statistical significance. The x-axis denotes the -$\log_{10}$(P) value [Fisher's exact test]. The figure was produced using Metascape (https://metascape.org/gp/index.html#/main/step1).

Supplementary table 1: Pathway-based burden test association analysis.

Pathway	*Variant Group*	*N° of Genes*	*N° of Variants*	*MAC*	*MAC in cases*	*MAC in controls*	*P-value*
ADIPOGENESIS	Group 1	200	100	177	16	161	0.053597
ADIPOGENESIS	Group 2	200	699	1346	138	1208	0.30979
ADIPOGENESIS	Group 3	200	2533	5856	678	5178	0.70878
ALLOGRAFT_REJECTION	Group 1	200	81	111	13	98	0.079106
ALLOGRAFT_REJECTION	Group 2	200	399	663	96	567	0.013383
ALLOGRAFT_REJECTION	Group 3	200	2185	5135	649	4486	0.53371
ANDROGEN_RESPONSE	Group 1	101	26	28	6	22	0.11991
ANDROGEN_RESPONSE	Group 2	101	232	374	48	326	0.78658
ANDROGEN_RESPONSE	Group 3	101	1135	2633	325	2308	0.81085
ANGIOGENESIS	Group 1	36	13	13	1	12	0.99984
ANGIOGENESIS	Group 2	36	133	284	42	242	0.0084621
ANGIOGENESIS	Group 3	36	571	1182	153	1029	0.29439
APICAL_JUNCTION	Group 1	200	100	138	13	125	0.94635
APICAL_JUNCTION	Group 2	200	738	1330	158	1172	0.71178
APICAL_JUNCTION	Group 3	200	3473	7937	934	7003	0.66608
APICAL_SURFACE	Group 1	44	29	38	3	35	0.99259
APICAL_SURFACE	Group 2	44	211	431	55	376	0.10663
APICAL_SURFACE	Group 3	44	903	2247	287	1960	0.3908
APOPTOSIS	Group 1	161	50	103	19	84	0.11384
APOPTOSIS	Group 2	161	356	670	90	580	0.13803
APOPTOSIS	Group 3	161	1688	3649	434	3215	0.4238
BILE_ACID_METABOLISM	Group 1	112	76	117	14	103	0.16825
BILE_ACID_METABOLISM	Group 2	112	600	1158	155	1003	0.0055858
BILE_ACID_METABOLISM	Group 3	112	1823	4231	510	3721	0.10503
CHOLESTEROL_HOMEOSTASIS	Group 1	74	28	52	6	46	0.58155
CHOLESTEROL_HOMEOSTASIS	Group 2	74	204	331	38	293	0.30755
CHOLESTEROL_HOMEOSTASIS	Group 3	74	851	1874	218	1656	0.2305
COAGULATION	Group 1	138	82	153	16	137	0.21254
COAGULATION	Group 2	138	564	1185	150	1035	0.11953
COAGULATION	Group 3	138	2165	5139	614	4525	0.48702
COMPLEMENT	Group 1	200	102	180	26	154	0.098689
COMPLEMENT	Group 2	200	637	1353	161	1192	0.57308
COMPLEMENT	Group 3	200	2912	6665	800	5865	0.32635
DNA_REPAIR	Group 1	150	46	92	12	80	0.38703
DNA_REPAIR	Group 2	150	261	459	51	408	0.59405
DNA_REPAIR	Group 3	150	1370	2797	337	2460	0.7788
E2F_TARGETS	Group 1	200	72	130	12	118	0.81999
E2F_TARGETS	Group 2	200	425	888	93	795	0.92595
E2F_TARGETS	Group 3	200	2714	6243	698	5545	0.8437
EPITHELIAL_MESENCHYMAL_ TRANSITION	Group 1	200	84	114	13	101	0.48466
EPITHELIAL_MESENCHYMAL_ TRANSITION	Group 2	200	1051	2154	271	1883	0.15864

EPITHELIAL_MESENCHYMAL_ TRANSITION	Group 3	200	3991	9239	1070	8169	0.67266
ESTROGEN_RESPONSE_EARLY	Group 1	200	111	145	15	130	0.75707
ESTROGEN_RESPONSE_EARLY	Group 2	200	745	1343	148	1195	0.60673
ESTROGEN_RESPONSE_EARLY	Group 3	200	3600	8135	931	7204	0.10273
ESTROGEN_RESPONSE_LATE	Group 1	200	109	157	13	144	0.89665
ESTROGEN_RESPONSE_LATE	Group 2	200	748	1417	166	1251	0.14493
ESTROGEN_RESPONSE_LATE	Group 3	200	3056	7276	809	6467	0.4808
FATTY_ACID_METABOLISM	Group 1	158	70	145	15	130	0.30315
FATTY_ACID_METABOLISM	Group 2	158	572	1189	144	1045	0.052322
FATTY_ACID_METABOLISM	Group 3	158	1621	3786	452	3334	0.044604
G2M_CHECKPOINT	Group 1	200	79	150	7	143	0.97876
G2M_CHECKPOINT	Group 2	200	367	629	59	570	0.71446
G2M_CHECKPOINT	Group 3	200	2977	6545	705	5840	0.86092
GLYCOLYSIS	Group 1	200	84	105	8	97	0.85479
GLYCOLYSIS	Group 2	200	801	1559	197	1362	0.14
GLYCOLYSIS	Group 3	200	2603	5614	669	4945	0.047341
HEDGEHOG_SIGNALING	Group 1	36	13	14	0	14	0.99995
HEDGEHOG_SIGNALING	Group 2	36	155	315	39	276	0.23363
HEDGEHOG_SIGNALING	Group 3	36	701	1477	164	1313	0.91087
HEME_METABOLISM	Group 1	200	74	116	9	107	0.7488
HEME_METABOLISM	Group 2	200	606	1254	158	1096	0.41344
HEME_METABOLISM	Group 3	200	2571	5859	685	5174	0.74589
HYPOXIA	Group 1	200	62	90	10	80	0.7515
HYPOXIA	Group 2	200	598	1089	155	934	0.091215
HYPOXIA	Group 3	200	2277	5070	628	4442	0.56509
IL2_STAT5_SIGNALING	Group 1	199	55	106	16	90	0.22451
IL2_STAT5_SIGNALING	Group 2	199	553	1130	143	987	0.070998
IL2_STAT5_SIGNALING	Group 3	199	2908	6859	825	6034	0.11388
IL6_JAK_STAT3_SIGNALING	Group 1	87	35	55	9	46	0.090138
IL6_JAK_STAT3_SIGNALING	Group 2	87	130	227	34	193	0.7033
IL6_JAK_STAT3_SIGNALING	Group 3	87	959	2342	275	2067	0.87492
INFLAMMATORY_RESPONSE	Group 1	200	86	203	16	187	0.24034
INFLAMMATORY_RESPONSE	Group 2	200	487	947	114	833	0.04343
INFLAMMATORY_RESPONSE	Group 3	200	2487	6205	715	5490	0.55236
INTERFERON_ALPHA_RESPONSE	Group 1	97	62	148	16	132	0.53247
INTERFERON_ALPHA_RESPONSE	Group 2	97	192	406	51	355	0.14662
INTERFERON_ALPHA_RESPONSE	Group 3	97	1338	2893	351	2542	0.32155
INTERFERON_GAMMA_RESPONSE	Group 1	200	93	232	27	205	0.74171
INTERFERON_GAMMA_RESPONSE	Group 2	200	387	757	93	664	0.087301
INTERFERON_GAMMA_RESPONSE	Group 3	200	2781	6280	694	5586	0.13513
KRAS_SIGNALING_DN	Group 1	200	109	206	22	184	0.51029
KRAS_SIGNALING_DN	Group 2	200	976	1860	189	1671	0.50251
KRAS_SIGNALING_DN	Group 3	200	3534	8238	931	7307	0.36804
KRAS_SIGNALING_UP	Group 1	200	93	162	16	146	0.77861
KRAS_SIGNALING_UP	Group 2	200	491	977	111	866	0.061947

KRAS_SIGNALING_UP	Group 3	200	2717	6465	781	5684	0.057307
MITOTIC_SPINDLE	Group 1	199	95	139	12	127	0.13073
MITOTIC_SPINDLE	Group 2	199	631	1104	126	978	0.57208
MITOTIC_SPINDLE	Group 3	199	4834	10515	1202	9313	0.33896
MTORC1_SIGNALING	Group 1	200	40	75	10	65	0.32612
MTORC1_SIGNALING	Group 2	200	431	851	107	744	0.48424
MTORC1_SIGNALING	Group 3	200	1672	3560	388	3172	0.77446
MYC_TARGETS_V1	Group 1	200	21	25	3	22	0.47229
MYC_TARGETS_V1	Group 2	200	209	411	46	365	0.47004
MYC_TARGETS_V1	Group 3	200	1114	2267	256	2011	0.89581
MYC_TARGETS_V2	Group 1	58	25	39	3	36	0.99948
MYC_TARGETS_V2	Group 2	58	124	200	25	175	0.65732
MYC_TARGETS_V2	Group 3	58	1023	2432	309	2123	0.014792
MYOGENESIS	Group 1	200	135	200	27	173	0.0073571
MYOGENESIS	Group 2	200	1264	2402	297	2105	0.18199
MYOGENESIS	Group 3	200	3924	9023	1053	7970	0.1848
NOTCH_SIGNALING	Group 1	32	2	4	0	4	0.9962
NOTCH_SIGNALING	Group 2	32	122	243	27	216	0.18963
NOTCH_SIGNALING	Group 3	32	416	982	105	877	0.13235
OXIDATIVE_PHOSPHORYLATION	Group 1	200	50	65	6	59	0.010443
OXIDATIVE_PHOSPHORYLATION	Group 2	200	534	1049	106	943	0.057816
OXIDATIVE_PHOSPHORYLATION	Group 3	200	1479	3212	365	2847	0.16814
P53_PATHWAY	Group 1	200	66	75	12	63	0.22739
P53_PATHWAY	Group 2	200	438	754	95	659	0.98613
P53_PATHWAY	Group 3	200	2291	5075	593	4482	0.925
PANCREAS_BETA_CELLS	Group 1	40	16	38	5	33	0.93289
PANCREAS_BETA_CELLS	Group 2	40	121	241	36	205	0.43031
PANCREAS_BETA_CELLS	Group 3	40	327	711	91	620	0.65681
PEROXISOME	Group 1	104	44	62	9	53	0.022
PEROXISOME	Group 2	104	435	788	102	686	0.2558
PEROXISOME	Group 3	104	1252	2754	356	2398	0.45773
PI3K_AKT_MTOR_SIGNALING	Group 1	105	25	28	5	23	0.015295
PI3K_AKT_MTOR_SIGNALING	Group 2	105	258	422	45	377	0.94794
PI3K_AKT_MTOR_SIGNALING	Group 3	105	1008	2150	250	1900	0.66075
PROTEIN_SECRETION	Group 1	96	19	21	2	19	0.99986
PROTEIN_SECRETION	Group 2	96	246	470	56	414	0.6299
PROTEIN_SECRETION	Group 3	96	1105	2286	295	1991	0.45946
REACTIVE_OXYGEN_SPECIES_PATHWAY	Group 1	49	21	50	10	40	0.01161
REACTIVE_OXYGEN_SPECIES_PATHWAY	Group 2	49	156	265	45	220	0.010461
REACTIVE_OXYGEN_SPECIES_PATHWAY	Group 3	49	486	928	140	788	0.0018168
SPERMATOGENESIS	Group 1	135	67	107	9	98	0.98408
SPERMATOGENESIS	Group 2	135	404	815	91	724	0.051371
SPERMATOGENESIS	Group 3	135	1787	3886	434	3452	0.62767
TGF_BETA_SIGNALING	Group 1	54	5	5	0	5	0.99182
TGF_BETA_SIGNALING	Group 2	54	138	200	26	174	0.32687

TGF_BETA_SIGNALING	Group 3	54	783	1505	165	1340	0.76929
TNFA_SIGNALING_VIA_NFKB	Group 1	200	53	111	12	99	0.88188
TNFA_SIGNALING_VIA_NFKB	Group 2	200	335	553	72	481	0.39978
TNFA_SIGNALING_VIA_NFKB	Group 3	200	2029	4597	528	4069	0.94629
UNFOLDED_PROTEIN_RESPONSE	Group 1	113	46	51	4	47	0.98454
UNFOLDED_PROTEIN_RESPONSE	Group 2	113	239	440	48	392	0.52281
UNFOLDED_PROTEIN_RESPONSE	Group 3	113	1223	2687	295	2392	0.14563
UV_RESPONSE_DN	Group 1	144	31	40	4	36	0.99581
UV_RESPONSE_DN	Group 2	144	534	1022	123	899	0.016803
UV_RESPONSE_DN	Group 3	144	2616	5758	669	5089	0.30006
UV_RESPONSE_UP	Group 1	158	39	80	7	73	0.6929
UV_RESPONSE_UP	Group 2	158	390	706	87	619	0.26763
UV_RESPONSE_UP	Group 3	158	1500	3056	350	2706	0.69499
WNT_BETA_CATENIN_SIGNALING	Group 1	42	7	8	2	6	0.98114
WNT_BETA_CATENIN_SIGNALING	Group 2	42	179	372	43	329	0.76465
WNT_BETA_CATENIN_SIGNALING	Group 3	42	708	1504	161	1343	0.51842
XENOBIOTIC_METABOLISM	Group 1	200	125	239	30	209	0.0049005
XENOBIOTIC_METABOLISM	Group 2	200	832	1481	210	1271	0.000015583
XENOBIOTIC_METABOLISM	Group 3	200	2606	5709	692	5017	0.040359

All association analyses (for variants included in Group 1, 2, or 3) for all Gene Set Enrichment Analysis (GSEA)[19] gene ontology pathways are shown. N cases = 215; N controls = 1,755. Abbreviations: MAC, minor allele counts; N°, number.

Supplementary table 2: Demographic and clinical features of 59 patients selected for RNA-seq analysis

Available as Supplementary_table2.xlsx file

Supplementary table 3: Significant differentially expressed genes.

Upregulated genes (hospitalized patients as reference)				
**Gene**	**baseMean**	**log2FoldChange**	**lfcSE**	***P-value (FDR)***
CMTM5	53.56	1.41	0.31	4.51×10^{-05}
GSN	56.66	1.12	0.26	2.77×10^{-04}
ENOSF1	140.83	1.11	0.27	3.10×10^{-04}
SLC24A3	14.35	1.36	0.37	3.10×10^{-04}
ITGA2B	391.46	1.28	0.34	4.30×10^{-04}
TSPAN33	27.01	1.19	0.32	6.39×10^{-04}
LCN2	35.41	1.24	0.35	6.39×10^{-04}
VSIG2	195.79	1.11	0.3	6.48×10^{-04}
ESAM	18.07	1.06	0.29	6.48×10^{-04}
PITPNM2	44.73	0.85	0.23	6.48×10^{-04}
ATP2A3	185.25	0.84	0.22	6.48×10^{-04}
ALOX12-AS1	51.89	1.1	0.32	6.48×10^{-04}
TADA3	56.55	0.65	0.17	6.48×10^{-04}
AGPAT1	154.2	0.97	0.26	6.48×10^{-04}
DNAH11	32.22	1.11	0.31	6.48×10^{-04}
TLN1	595.32	0.95	0.26	6.48×10^{-04}
PTGS1	118.43	1.08	0.3	6.48×10^{-04}
TREML1	58.51	1.08	0.31	6.83×10^{-04}
PPP2R4	17.34	0.85	0.24	8.76×10^{-04}
RAB1B	118.07	0.77	0.22	9.26×10^{-04}
STOM	78.9	1.03	0.3	9.26×10^{-04}
ACTB	2141.66	0.72	0.21	1.05×10^{-03}
LOC100506606	69.79	1.07	0.33	1.06×10^{-03}
SLC39A3	22.35	0.95	0.29	1.14×10^{-03}
SLC44A2	158.23	0.75	0.22	1.15×10^{-03}
MCU	21.54	0.87	0.26	1.22×10^{-03}
ITGA9	12.2	1.03	0.32	1.22×10^{-03}
F11R	28.18	0.76	0.23	1.28×10^{-03}
MARCH2	52.78	1.02	0.32	1.28×10^{-03}
SLC2A5	11.62	1.11	0.43	1.39×10^{-03}
LTBP1	28.42	1.01	0.33	1.39×10^{-03}
FAM189B	7.6	0.96	0.31	1.51×10^{-03}
LAT	19.08	0.93	0.3	1.51×10^{-03}
PPBP	303.75	1.05	0.37	1.51×10^{-03}
PEAR1	18.29	1.07	0.39	1.53×10^{-03}
MTURN	78.07	0.95	0.31	1.66×10^{-03}
ACTN1	81.22	0.77	0.25	2.08×10^{-03}
ITGB5	14.96	1	0.37	2.33×10^{-03}
GSN-AS1	148.14	0.79	0.26	2.33×10^{-03}
GAS2L1	15.78	1.01	0.38	2.49×10^{-03}
MAP1A	20.4	1	0.39	2.56×10^{-03}
MAVS	33.92	0.69	0.23	2.79×10^{-03}
PARVB	19.04	0.92	0.33	2.79×10^{-03}
LY6G6E	180.26	0.92	0.33	2.79×10^{-03}
RBPMS2	6	0.98	0.53	2.83×10^{-03}
CYB5R3	110.61	0.85	0.3	2.87×10^{-03}
NCAPD2	11.42	0.83	0.29	3.10×10^{-03}
PGLYRP1	22.46	0.98	0.43	3.27×10^{-03}
ITGB3	245.07	0.94	0.38	3.85×10^{-03}
AGBL5	665.75	0.78	0.29	4.57×10^{-03}
GP9	10.26	0.94	0.42	4.78×10^{-03}
MYLK	38.89	0.8	0.3	4.79×10^{-03}
RAB6B	32.3	0.83	0.32	4.91×10^{-03}
PCSK6	17.6	0.91	0.4	5.12×10^{-03}
GPI	43.39	0.61	0.21	5.18×10^{-03}
OST4	126.02	0.84	0.33	5.18×10^{-03}
MLC1	7.37	0.92	0.43	5.18×10^{-03}
ZNF185	41.88	0.73	0.27	5.18×10^{-03}
PKMYT1	50.95	0.74	0.28	5.25×10^{-03}
LOC101928909	20.34	0.81	0.32	5.32×10^{-03}
F2R	17.75	0.8	0.32	5.44×10^{-03}

RXFP2	13.77	0.89	0.39	5.63×10^{-03}
LINC00094	63.68	0.52	0.17	5.68×10^{-03}
DGKG	6.17	0.87	0.38	5.75×10^{-03}
PF4	137.31	0.82	0.34	5.99×10^{-03}
C1orf198	15.39	0.82	0.34	6.00×10^{-03}
SEPN1	31.42	0.66	0.25	6.29×10^{-03}
TAL1	30.99	0.85	0.38	6.29×10^{-03}
KIAA0125	4.48	0.87	0.42	6.29×10^{-03}
MYL9	62.97	0.85	0.37	6.29×10^{-03}
PCNA-AS1	64.18	0.71	0.28	6.38×10^{-03}
UFD1L	11.03	0.55	0.2	6.38×10^{-03}
VSTM1	7.5	0.85	0.4	6.53×10^{-03}
TTC7B	10.13	0.86	0.43	6.66×10^{-03}
PDLIM1	34.17	0.8	0.34	6.71×10^{-03}
ATP5D	10.39	0.6	0.22	6.72×10^{-03}
GNAZ	16.19	0.82	0.37	6.77×10^{-03}
CENPT	26.64	0.71	0.29	7.20×10^{-03}
ABCC4	28.43	0.84	0.41	7.33×10^{-03}
CCL5	93.48	0.71	0.29	7.33×10^{-03}
WNT11	5.72	0.81	0.49	7.35×10^{-03}
DDX11L10	12.02	0.84	0.43	7.45×10^{-03}
G6PD	33.71	0.54	0.2	7.45×10^{-03}
LOC101928445	334.88	0.72	0.3	7.49×10^{-03}
YIF1B	154.95	0.83	0.41	7.59×10^{-03}
MYH9	671.76	0.52	0.19	7.63×10^{-03}
RYR2	15.32	0.82	0.45	7.73×10^{-03}
HIST1H3B	6.87	0.82	0.43	8.68×10^{-03}
CAPN1	100.62	0.63	0.25	8.79×10^{-03}
RTN3	150.38	0.57	0.22	9.04×10^{-03}
DAAM1	10.13	0.77	0.36	9.23×10^{-03}
FADS2	31.1	0.73	0.33	9.51×10^{-03}
CLCN4	4.31	0.77	0.47	9.81×10^{-03}
SLC44A1	13.95	0.76	0.36	9.97×10^{-03}
MS4A3	12.73	0.69	0.52	1.01×10^{-02}
ACTG1	598.29	0.57	0.23	1.01×10^{-02}
AUNIP	56.5	0.64	0.27	1.03×10^{-02}
VCL	179.18	0.54	0.2	1.03×10^{-02}
SUSD1	20.06	0.67	0.29	1.03×10^{-02}
PYCR2	15.19	0.72	0.33	1.03×10^{-02}
CEACAM6	21.41	0.68	0.52	1.03×10^{-02}
NRGN	601.54	0.7	0.31	1.06×10^{-02}
CEACAM8	35.99	0.76	0.46	1.10×10^{-02}
LINC01011	16.84	0.75	0.37	1.13×10^{-02}
THCAT158	6.53	0.75	0.47	1.14×10^{-02}
PLTP	279.06	0.53	0.2	1.17×10^{-02}
CDK2AP1	9.37	0.7	0.33	1.22×10^{-02}
SELP	15.3	0.74	0.38	1.23×10^{-02}
TYMSOS	21.97	0.72	0.35	1.24×10^{-02}
YWHAH	40.85	0.66	0.3	1.24×10^{-02}
CTSG	7.46	0.54	0.52	1.33×10^{-02}
SMOX	145.42	0.72	0.37	1.34×10^{-02}
SH3BGRL3	94.17	0.51	0.2	1.37×10^{-02}
MED12L	22.97	0.64	0.3	1.38×10^{-02}
TPST2	19.55	0.6	0.26	1.39×10^{-02}
ENDOD1	12.72	0.73	0.41	1.40×10^{-02}
TRAPPC1	64.53	0.53	0.22	1.40×10^{-02}
LTF	343.01	0.72	0.43	1.40×10^{-02}
CRAT	18.87	0.66	0.32	1.40×10^{-02}
RUFY1	78.29	0.56	0.24	1.41×10^{-02}
VIPAS39	6.76	0.64	0.3	1.47×10^{-02}
CLTCL1	4.01	0.7	0.37	1.47×10^{-02}
CCND3	66.66	0.54	0.22	1.47×10^{-02}
LOC100507424	9.74	0.71	0.39	1.51×10^{-02}
CTB-113P19.1	134.95	0.62	0.29	1.53×10^{-02}
C6orf25	95.65	0.65	0.32	1.53×10^{-02}

NDUFAF3	13.23	0.63	0.3	1.54×10^{-02}
PROS1	15.84	0.71	0.43	1.55×10^{-02}
GATA1	8.42	0.64	0.48	1.55×10^{-02}
PTPRJ	91.97	0.52	0.22	1.59×10^{-02}
ANP32B	43.65	0.63	0.31	1.63×10^{-02}
TALDO1	60.2	0.54	0.23	1.68×10^{-02}
LAMTOR1	76.32	0.54	0.23	1.69×10^{-02}
LOC100133286	68.84	0.57	0.25	1.69×10^{-02}
CLU	166.11	0.61	0.3	1.77×10^{-02}
ZYX	129.45	0.5	0.21	1.88×10^{-02}
ATP13A2	13.78	0.5	0.22	1.88×10^{-02}
PRTN3	5.61	0.55	0.49	1.88×10^{-02}
RRM2	4.9	0.66	0.36	1.88×10^{-02}
GPX1	212.47	0.59	0.28	1.94×10^{-02}
LIPE	10.66	0.54	0.24	1.94×10^{-02}
DNAH10	22.15	0.58	0.28	1.95×10^{-02}
CHADL	18.12	0.55	0.25	1.96×10^{-02}
RN7SL1	8538.99	0.61	0.31	1.99×10^{-02}
INF2	15.09	0.63	0.33	2.03×10^{-02}
BMP6	171.87	0.58	0.29	2.08×10^{-02}
RBX1	8.53	0.62	0.33	2.09×10^{-02}
WBP2	62.33	0.62	0.33	2.11×10^{-02}
TBXA2R	8.91	0.65	0.39	2.14×10^{-02}
COPS5	6.61	0.59	0.3	2.14×10^{-02}
UBL4A	16.05	0.6	0.31	2.14×10^{-02}
OAZ1	147.09	0.58	0.29	2.14×10^{-02}
ACOT8	9.81	0.54	0.25	2.14×10^{-02}
BEND2	7.85	0.62	0.44	2.29×10^{-02}
CD151	26.01	0.55	0.27	2.29×10^{-02}
MPO	64.26	0.63	0.42	2.32×10^{-02}
EHD3	33.53	0.6	0.32	2.32×10^{-02}
RNY4	19.18	0.62	0.44	2.32×10^{-02}
CTDSPL	13.08	0.63	0.38	2.38×10^{-02}
AQP10	7.64	0.61	0.44	2.46×10^{-02}
H2AFX	6.48	0.61	0.36	2.52×10^{-02}
HMGN2	20.47	0.51	0.25	2.70×10^{-02}
SLC25A45	22.03	0.58	0.31	2.70×10^{-02}
CDCA7L	8.12	0.61	0.37	2.70×10^{-02}
FAM63A	25.23	0.53	0.27	2.73×10^{-02}
HMMR-AS1	24.63	0.61	0.39	2.74×10^{-02}
PROSER2-AS1	19.9	0.55	0.29	2.81×10^{-02}
LPO	5.27	0.57	0.44	2.88×10^{-02}
MFSD2B	9.48	0.58	0.43	2.88×10^{-02}
MCUR1	8.67	0.57	0.33	2.88×10^{-02}
TCN1	8.04	0.55	0.45	2.90×10^{-02}
ABCC3	28.21	0.55	0.3	2.90×10^{-02}
AZU1	19.62	0.54	0.45	2.90×10^{-02}
GUCY1B3	13.78	0.58	0.35	2.97×10^{-02}
AVL9	37.68	0.55	0.3	3.05×10^{-02}
NID1	19.85	0.58	0.37	3.12×10^{-02}
HSPA12B	8.65	0.57	0.33	3.12×10^{-02}
TPX2	5.89	0.58	0.41	3.13×10^{-02}
RN7SL2	33295.6	0.54	0.3	3.14×10^{-02}
TSPAN2	4.05	0.57	0.42	3.14×10^{-02}
PRUNE	13.02	0.53	0.29	3.14×10^{-02}
ACRBP	42.06	0.54	0.3	3.19×10^{-02}
SPINT2	10.09	0.57	0.37	3.19×10^{-02}
MEMO1	7.97	0.51	0.27	3.19×10^{-02}
RCAN1	7.79	0.57	0.41	3.19×10^{-02}
GRAP2	61.13	0.55	0.32	3.19×10^{-02}
ITGA9-AS1	4.54	0.56	0.42	3.19×10^{-02}
TMEM185A	7.82	0.57	0.36	3.20×10^{-02}
PAQR7	8.9	0.56	0.34	3.22×10^{-02}
TUBA8	17.83	0.56	0.33	3.22×10^{-02}
SPARC	212.05	0.54	0.31	3.22×10^{-02}

F13A1	140.05	0.54	0.31	3.22×10^{-02}
RTN1	5.11	0.56	0.41	3.22×10^{-02}
SAMD14	13.43	0.57	0.39	3.25×10^{-02}
KLHDC8B	4.46	0.55	0.41	3.36×10^{-02}
PLXNB3	7.2	0.55	0.42	3.36×10^{-02}
HDGF	209.2	0.53	0.31	3.44×10^{-02}
CSRP1	8.57	0.54	0.33	3.64×10^{-02}
GUCY1A3	7.24	0.54	0.4	3.75×10^{-02}
DPY19L1	6.14	0.54	0.35	3.79×10^{-02}
MSRB3	3.73	0.51	0.42	3.81×10^{-02}
KIF3C	4.87	0.52	0.39	4.10×10^{-02}
SLC39A4	4.45	0.53	0.36	4.12×10^{-02}
ABLIM3	18.9	0.52	0.33	4.13×10^{-02}
C9orf69	4.72	0.52	0.35	4.31×10^{-02}
PBX1	12.95	0.51	0.39	4.47×10^{-02}
TERC	9.08	0.51	0.36	4.52×10^{-02}
SLC35B4	4.07	0.51	0.35	4.55×10^{-02}
CTTN	17.75	0.5	0.35	4.62×10^{-02}
MYBL2	15.89	0.5	0.34	4.65×10^{-02}
PCYOX1L	3.34	0.5	0.38	4.65×10^{-02}
Downregulated genes (hospitalized patients as reference)				
**Gene**	**baseMean**	**log2FoldChange**	**lfcSE**	***P-value (FDR)***
IL1RN	45.3	-1.48	0.33	4.51×10^{-05}
ARID4B	27.42	-0.65	0.17	6.48×10^{-04}
RASA1	474.32	-0.82	0.21	6.48×10^{-04}
EYS	33.32	-0.79	0.21	6.48×10^{-04}
CEP83	39.64	-1.07	0.33	9.62×10^{-04}
LOC101929512	33.39	-1.07	0.34	1.14×10^{-03}
BACH1	57.34	-0.67	0.2	1.22×10^{-03}
RNF175	67.76	-1	0.31	1.22×10^{-03}
TRAPPC13	43.47	-0.86	0.26	1.37×10^{-03}
MARCKS	89.31	-0.85	0.26	1.39×10^{-03}
ZNF512B	21.07	-1.04	0.36	1.50×10^{-03}
ATP2B1	30.81	-0.8	0.25	1.51×10^{-03}
ZC3H12A	65.6	-0.91	0.29	1.55×10^{-03}
SDCBP	107.41	-0.58	0.17	1.93×10^{-03}
AP1AR	15.31	-0.94	0.35	2.83×10^{-03}
RANBP2	34.53	-0.7	0.23	2.87×10^{-03}
CHST7	5.73	-0.9	0.33	3.18×10^{-03}
PGM3	43.62	-0.59	0.19	3.22×10^{-03}
LINC01531	4.76	-0.8	0.72	3.27×10^{-03}
ATP13A3	21.15	-0.65	0.22	3.69×10^{-03}
LINC01004	44.41	-0.77	0.27	3.93×10^{-03}
HCAR3	9.35	-0.95	0.44	4.29×10^{-03}
CD300E	118.98	-0.79	0.3	4.75×10^{-03}
TDRD6	12.95	-0.88	0.36	4.91×10^{-03}
LPAR6	25.76	-0.69	0.25	4.98×10^{-03}
STX17	38.07	-0.59	0.2	4.98×10^{-03}
SGTB	9.37	-0.67	0.24	5.19×10^{-03}
FAM98B	20.33	-0.83	0.33	5.32×10^{-03}
C10orf55	182.76	-0.88	0.49	5.63×10^{-03}
NFKBIZ	110.83	-0.68	0.25	5.63×10^{-03}
LINC01108	16.83	-0.9	0.45	5.63×10^{-03}
MARCKSL1	57.6	-0.66	0.24	5.64×10^{-03}
HIF1A	151.47	-0.64	0.23	5.64×10^{-03}
SERPINB9P1	22.92	-0.85	0.36	5.64×10^{-03}
SLC24A5	9.27	-0.83	0.35	5.83×10^{-03}
SAMSN1	26.05	-0.68	0.26	6.29×10^{-03}
PHACTR1	19.63	-0.74	0.29	6.29×10^{-03}
GNS	76.8	-0.7	0.27	6.32×10^{-03}
NAB2	46.19	-0.7	0.27	6.38×10^{-03}
ZNF267	14.31	-0.68	0.26	6.38×10^{-03}
EPGN	11.83	-0.86	0.41	6.38×10^{-03}
DDX60L	26.69	-0.78	0.32	6.38×10^{-03}
LCP2	99.35	-0.57	0.2	6.51×10^{-03}

CTNNB1	58.7	-0.52	0.18	7.33 × 10^{-03}
PTCH2	10.54	-0.84	0.44	7.35 × 10^{-03}
TAF1C	36.9	-0.71	0.29	7.35 × 10^{-03}
KCNS1	13.45	-0.81	0.38	7.45 × 10^{-03}
GPRIN3	25.81	-0.61	0.23	7.48 × 10^{-03}
TM9SF1	45.15	-0.75	0.34	8.79 × 10^{-03}
COPZ1	127.33	-0.7	0.3	8.86 × 10^{-03}
NFKBID	36.66	-0.71	0.32	9.79 × 10^{-03}
RNF19B	45.2	-0.67	0.29	1.03 × 10^{-02}
NID2	37.1	-0.53	0.2	1.03 × 10^{-02}
TGFBI	17.77	-0.64	0.27	1.03 × 10^{-02}
OSGIN2	84.72	-0.77	0.38	1.04 × 10^{-02}
ICAM1	97.2	-0.72	0.33	1.06 × 10^{-02}
TRAF6	12.89	-0.56	0.22	1.16 × 10^{-02}
HIF1A-AS2	669.57	-0.61	0.26	1.16 × 10^{-02}
GADD45B	48.06	-0.62	0.27	1.23 × 10^{-02}
RASGEF1B	21.1	-0.64	0.28	1.23 × 10^{-02}
ERO1B	57.61	-0.66	0.3	1.24 × 10^{-02}
ZDHHC18	71.97	-0.65	0.29	1.26 × 10^{-02}
GBP3	9.23	-0.63	0.28	1.28 × 10^{-02}
MAP3K8	19.15	-0.52	0.21	1.34 × 10^{-02}
MN1	3.56	-0.69	0.49	1.34 × 10^{-02}
SLC35A1	18.57	-0.58	0.25	1.34 × 10^{-02}
NBN	27.23	-0.64	0.29	1.34 × 10^{-02}
OSM	34.91	-0.59	0.26	1.36 × 10^{-02}
GBP5	50.54	-0.74	0.43	1.36 × 10^{-02}
PFN2	12.24	-0.71	0.36	1.37 × 10^{-02}
C5orf58	394.79	-0.56	0.23	1.37 × 10^{-02}
AHCTF1	20.83	-0.57	0.24	1.40 × 10^{-02}
THRA1/BTR	34.42	-0.6	0.27	1.40 × 10^{-02}
HAVCR2	11.44	-0.63	0.28	1.40 × 10^{-02}
GPR65	9.18	-0.67	0.32	1.44 × 10^{-02}
TENM1	202.86	-0.63	0.29	1.47 × 10^{-02}
GNRH1	6.66	-0.69	0.36	1.51 × 10^{-02}
BTBD19	12.65	-0.63	0.3	1.54 × 10^{-02}
LRIF1	67.81	-0.52	0.21	1.58 × 10^{-02}
LOC101927131	23.64	-0.67	0.47	1.59 × 10^{-02}
EPB41L5	22.43	-0.7	0.43	1.59 × 10^{-02}
STARD4	46.3	-0.56	0.25	1.61 × 10^{-02}
TAGAP	69.58	-0.54	0.24	1.68 × 10^{-02}
PARP14	31.75	-0.57	0.26	1.73 × 10^{-02}
CD83	31.17	-0.62	0.31	1.80 × 10^{-02}
HDAC1	71.26	-0.53	0.23	1.84 × 10^{-02}
DKC1	52.11	-0.6	0.29	1.86 × 10^{-02}
OXNAD1	32.08	-0.53	0.24	1.95 × 10^{-02}
DMXL2	40.08	-0.52	0.23	1.96 × 10^{-02}
NAMPT	254.93	-0.59	0.29	2.01 × 10^{-02}
ERMP1	23.62	-0.59	0.29	2.05 × 10^{-02}
RALGDS	72.2	-0.53	0.25	2.07 × 10^{-02}
MICAL3	87.78	-0.65	0.41	2.11 × 10^{-02}
HCAR2	5.86	-0.63	0.44	2.14 × 10^{-02}
BBIP1	373.38	-0.52	0.24	2.27 × 10^{-02}
NINJ1	66.66	-0.5	0.23	2.29 × 10^{-02}
GSTO2	8.56	-0.63	0.37	2.29 × 10^{-02}
CPD	33.36	-0.51	0.23	2.29 × 10^{-02}
AHR	35.33	-0.54	0.26	2.29 × 10^{-02}
MUC5AC	16.94	-0.63	0.41	2.32 × 10^{-02}
N4BP1	37.59	-0.58	0.3	2.32 × 10^{-02}
NAALAD2	8.73	-0.58	0.3	2.34 × 10^{-02}
IL1B	40.63	-0.63	0.4	2.34 × 10^{-02}
GPX3	103.52	-0.63	0.41	2.34 × 10^{-02}
TLR2	37.53	-0.5	0.23	2.37 × 10^{-02}
B3GNT5	13.57	-0.54	0.26	2.38 × 10^{-02}
PLN	17.86	-0.51	0.24	2.49 × 10^{-02}
APPL2	42.41	-0.51	0.24	2.51 × 10^{-02}

LAMB1	24.87	-0.54	0.27	2.54×10^{-02}
SLC5A3	6.04	-0.59	0.33	2.63×10^{-02}
PIK3IP1	313.26	-0.51	0.25	2.67×10^{-02}
LOC100506801	140.53	-0.58	0.33	2.73×10^{-02}
LOC653513	18.59	-0.52	0.26	2.73×10^{-02}
MIR1260B	59.29	-0.5	0.24	2.73×10^{-02}
BCL2L13	75.23	-0.59	0.33	2.73×10^{-02}
STRBP	7.76	-0.55	0.29	2.74×10^{-02}
ZNF12	7.79	-0.59	0.35	2.79×10^{-02}
LINC00173	7.64	-0.58	0.34	2.88×10^{-02}
TNFRSF8	12.65	-0.55	0.3	2.88×10^{-02}
LOC100130357	192.68	-0.56	0.31	3.01×10^{-02}
EIF2D	68.36	-0.55	0.3	3.04×10^{-02}
SLC1A2	30.47	-0.52	0.27	3.14×10^{-02}
GK5	6.06	-0.56	0.33	3.14×10^{-02}
RORA	34.65	-0.5	0.26	3.16×10^{-02}
COL4A2	15.35	-0.57	0.37	3.19×10^{-02}
CAMKK1	5.16	-0.57	0.38	3.19×10^{-02}
CDC42EP3	162.42	-0.56	0.35	3.32×10^{-02}
BCL2A1	36.96	-0.53	0.31	3.54×10^{-02}
TTC21A	32.85	-0.5	0.28	3.62×10^{-02}
SEPP1	8.66	-0.53	0.33	3.79×10^{-02}
LOC102723604	166.45	-0.54	0.35	3.79×10^{-02}
ARHGAP31-AS1	18.4	-0.53	0.32	3.83×10^{-02}
ESPN	3.4	-0.53	0.38	3.92×10^{-02}
KIRREL3	36.09	-0.52	0.33	3.92×10^{-02}
ERMN	10.87	-0.52	0.36	4.27×10^{-02}
ARHGEF26	6.15	-0.52	0.36	4.28×10^{-02}
FCGR3B	23.3	-0.51	0.38	4.47×10^{-02}

All significant differentially expressed genes are shown. Among upregulated genes, platelet-specific canonical markers are highlighted in red, megakaryocyte-specific marker is highlighted in yellow, and shared platelet/megakaryocyte markers are highlighted in light orange. N ICU-admitted = 35; N hospitalized = 24. Abbreviations: lfcSE, log2 fold change standard error; FDR: false discovery rate.

Supplementary table 4: Haplotype association analysis in the *LPO* locus.

A) Haplotype analysis: omnibus results

Window	*ChiSQ*	*DF*	*P*	*SNPs*
10	4.598	6	0.5963	chr17:58216599:T:C|chr17:58216780:C:T|chr17:58217547:G:A|chr17:58218250:A:C|chr17:58218340:C:A|chr17:58218875:C:G|chr17:58219007:G:T|chr17:58221146:C:T|chr17:58222905:C:T|**chr17:58222906:G:A**
9	4.87	6	0.5606	chr17:58216780:C:T|chr17:58217547:G:A|chr17:58218250:A:C|chr17:58218340:C:A|chr17:58218875:C:G|chr17:58219007:G:T|chr17:58221146:C:T|chr17:58222905:C:T|**chr17:58222906:G:A**|chr17:58222998:C:T
8	4.542	6	0.6037	chr17:58217547:G:A|chr17:58218250:A:C|chr17:58218340:C:A|chr17:58218875:C:G|chr17:58219007:G:T|chr17:58221146:C:T|chr17:58222905:C:T|**chr17:58222906:G:A**|chr17:58222998:C:T|chr17:58224432:A:G
7	4.597	5	0.467	chr17:58218250:A:C|chr17:58218340:C:A|chr17:58218875:C:G|chr17:58219007:G:T|chr17:58221146:C:T|chr17:58222905:C:T|**chr17:58222906:G:A**|chr17:58222998:C:T|chr17:58224432:A:G|chr17:58224686:G:A
6	11.87	6	0.06492	chr17:58218340:C:A|chr17:58218875:C:G|chr17:58219007:G:T|chr17:58221146:C:T |chr17:58222905:C:T|**chr17:58222906:G:A**|chr17:58222998:C:T|chr17:58224432:A:G|chr17:58224686:G:A|chr17:58225531:A:G
5	14.39	6	0.02553	chr17:58218875:C:G|chr17:58219007:G:T|chr17:58221146:C:T|chr17:58222905:C:T|**chr17:58222906:G:A**|chr17:58222998:C:T|chr17:58224432:A:G|chr17:58224686:G:A|chr17:58225531:A:G|chr17:58225690:G:A
4	15.41	6	0.01727	chr17:58219007:G:T|chr17:58221146:C:T|chr17:58222905:C:T|**chr17:58222906:G:A**|chr17:58222998:C:T|chr17:58224432:A:G|chr17:58224686:G:A|chr17:58225531:A:G|chr17:58225690:G:A|chr17:58225697:C:T
3	15.69	6	0.0155	chr17:58221146:C:T|chr17:58222905:C:T|**chr17:58222906:G:A**|chr17:58222998:C:T|chr17:58224432:A:G|chr17:58224686:G:A|chr17:58225531:A:G|chr17:58225690:G:A|chr17:58225697:C:T|chr17:58225825:G:A
2	15.84	7	0.02662	chr17:58222905:C:T|**chr17:58222906:G:A**|chr17:58222998:C:T|chr17:58224432:A:G|chr17:58224686:G:A|chr17:58225531:A:G|chr17:58225690:G:A|chr17:58225697:C:T|chr17:58225825:G:A|chr17:58225926:G:T
1	15.84	7	0.02662	**chr17:58222906:G:A**|chr17:58222998:C:T|chr17:58224432:A:G|chr17:58224686:G:A|chr17:58225531:A:G|chr17:58225690:G:A|chr17:58225697:C:T|chr17:58225825:G:A|chr17:58225926:G:T|chr17:58226006:A:G

The association analysis was performed with Plink v1.07, using the sliding-window approach. Haplotype windows on chromosome 17 encompassed the genomic region 58,216,599-58,226,006 (GRCh38). The "Window" column provides the sequential identifier for the analysis. The "SNPs" column lists the 10 variants included in each window. The rs57397900 polymorphism (chr17:58222906:G:A), to which all windows are anchored, is bolded. Uncorrected P values are reported.
ChiSQ, Chi-square statistic; DF, degrees of freedom for the test.

B) Association results for the individual haplotypes identified within the most significant window

Window	*Haplotype*	*Frequency in cases*	*Frequency in controls*	*ChiSQ*	*P*
3	CCGCAGAGCG	0.6980	0.6862	0.5857	0.4441
	CCGCGGAGTG	0.05508	0.07763	6.675	**0.009775**
	CCGCAGGGCG	0.03514	0.02085	8.274	**0.004022**
	TCACAAAGCG	0.02849	0.02785	0.01378	0.9066
	CTGTAGAGCA	0.09972	0.1097	0.9299	0.3349
	CCGCGGAATG	0.03039	0.02769	0.2414	0.6232
	CCACAGAGCG	0.05318	0.05009	0.1798	0.6715

ChiSQ, Chi-square statistic. Uncorrected P values are reported.

Supplementary table 5: Significant splicing events.

Available as Supplementary_table5.xlsx file

avg_inc_cases, average number of reads supporting the inclusion of the splicing event in the case group;

avg_inc_ctr, average number of reads supporting the inclusion of the splicing event in the control group;

avg_exc_cases, average number of reads supporting the exclusion of the splicing event in the case group;

avg_exc_ctr, average number of reads supporting the exclusion of the splicing event in the control group.

Supplementary table 6: Mean expression correlation of differentially alternatively spliced (DAG) transcription factors (TF) and their target genes.

Available as Supplementary_table6.xlsx file

Only DAG-TF with at least 2 DEG (differentially expressed genes) among their targets are shown.

mean_corr, mean correlation between transcription factor expression levels and its target genes expression levels;

mean_corr_hospital , mean correlation between transcription factor expression levels and its target genes expression levels only in hospitalized patients;

mean_corr_ICU, mean correlation between transcription factor expression levels and its target genes expression levels only in patients admitted to the ICU.

SUPPLEMENTARY REFERENCES

Purcell S, Neale B, Todd-Brown K, Thomas L, Ferreira MA, Bender D, et al. PLINK: a tool set for whole-genome association and population-based linkage analyses. *Am J Hum Genet.* 2007;**81:**559-75.

Degenhardt F, Ellinghaus D, Juzenas S, Lerga-Jaso J, Wendorff M, Maya-Miles D, et al. Detailed stratified GWAS analysis for severe COVID-19 in four European populations. *Hum Mol Genet.* 2022;**31:**3945-66.

Severe Covid-19 GWAS Group; Ellinghaus D, Degenhardt F, Bujanda L, Buti M, Albillos A, et al. Genomewide Association Study of Severe Covid-19 with Respiratory Failure. *N Engl J Med.* 2020;**383:**1522-34.

Myocardial Infarction Genetics Consortium; Kathiresan S, Voight BF, Purcell S, Musunuru K, Ardissino D, Mannucci PM, et al. Genome-wide association of early-onset myocardial infarction with single nucleotide polymorphisms and copy number variants. *Nat Genet.* 2009;**41**:334-41.
